# Bursting Neurons in the Hippocampal Formation Encode Features of LFP Rhythms

**DOI:** 10.3389/fncom.2016.00133

**Published:** 2016-12-26

**Authors:** Maria Constantinou, Soledad Gonzalo Cogno, Daniel H. Elijah, Emilio Kropff, John Gigg, Inés Samengo, Marcelo A. Montemurro

**Affiliations:** ^1^Faculty of Biology, Medicine and Health, The University of ManchesterManchester, UK; ^2^Centro Atómico Bariloche and Instituto BalseiroSan Carlos de Bariloche, Argentina; ^3^Leloir Institute, IIBBA-CONICETBuenos Aires, Argentina

**Keywords:** bursting, local field potential, subiculum, entorhinal cortex, information theory, neural coding

## Abstract

Burst spike patterns are common in regions of the hippocampal formation such as the subiculum and medial entorhinal cortex (MEC). Neurons in these areas are immersed in extracellular electrical potential fluctuations often recorded as the local field potential (LFP). LFP rhythms within different frequency bands are linked to different behavioral states. For example, delta rhythms are often associated with slow-wave sleep, inactivity and anesthesia; whereas theta rhythms are prominent during awake exploratory behavior and REM sleep. Recent evidence suggests that bursting neurons in the hippocampal formation can encode LFP features. We explored this hypothesis using a two-compartment model of a bursting pyramidal neuron driven by time-varying input signals containing spectral peaks at either delta or theta rhythms. The model predicted a neural code in which bursts represented the instantaneous value, phase, slope and amplitude of the driving signal both in their timing and size (spike number). To verify whether this code is employed *in vivo*, we examined electrophysiological recordings from the subiculum of anesthetized rats and the MEC of a behaving rat containing prevalent delta or theta rhythms, respectively. In both areas, we found bursting cells that encoded information about the instantaneous voltage, phase, slope and/or amplitude of the dominant LFP rhythm with essentially the same neural code as the simulated neurons. A fraction of the cells encoded part of the information in burst size, in agreement with model predictions. These results provide *in-vivo* evidence that the output of bursting neurons in the mammalian brain is tuned to features of the LFP.

## 1. Introduction

Bursts are groups of high frequency spikes followed by quiescent periods. In the mammalian brain, bursting activity has been observed in the cortex (Connors et al., 1982; McCormick et al., 1985), thalamus (Steriade et al., 1993; Guido and Weyand, 1995) and hippocampal formation (Kandel and Spencer, 1961; Ranck, 1973) among other regions. However, despite being ubiquitous, little is known about the specific role of bursts in information processing. From a dynamical point of view, bursts are not simply a sequence of individual spikes fired in rapid succession. They rather constitute a single dynamical event triggered and supported by the interplay between slow and fast currents underpinning the cell's membrane excitability (Izhikevich, 2010).

Bursting neurons have been identified in regions of the rodent hippocampal formation such as the subiculum (Sharp and Green, 1994; Gigg et al., 2000) and more recently in the medial entorhinal cortex (MEC) (Latuske et al., 2015). Both of these areas are important for processing hippocampal information (e.g., Hafting et al., 2005; Kim et al., 2012). The subiculum receives input from area CA1 and projects hippocampal output to cortical and subcortical areas (for reviews see O'Mara et al., 2001; Gigg, 2006) whereas the MEC receives cortical and subcortical input and projects to the hippocampus (Canto et al., 2008; Zhang et al., 2014).

Neurons are immersed in electrical potential oscillations that can be recorded in the extracellular milieu as the local field potential (LFP). The LFP reflects the sum of all transmembrane currents in the vicinity of the recording electrode (Logothetis, 2003; Buzsáki et al., 2012) with a predominant contribution from synaptic activity of populations of pyramidal neurons within a volume of neural tissue (Einevoll et al., 2007; Pettersen et al., 2008). Hence, extracellular oscillations usually contain information about the local network activity. Oscillations within specific frequency bands have been associated with a range of cognitive functions (Engel et al., 2001; Ward, 2003; Wang, 2010). For instance, in the hippocampal formation theta and gamma rhythms are involved in memory processing (Lisman and Idiart, 1995; Lisman, 2005) and spatial navigation (O'Keefe and Recce, 1993; Skaggs et al., 1996; McNaughton et al., 2006), whereas delta rhythms and slow oscillations are involved in memory consolidation (Mölle and Born, 2011; Rasch and Born, 2013; Buzsáki, 2015). In addition, LFP rhythms have been suggested to provide a time frame for neuronal interactions and organizing neuronal activity (Fries, 2005; Womelsdorf et al., 2007). Moreover, evidence from the monkey visual (Montemurro et al., 2008) and auditory cortices (Kayser et al., 2009) suggests that the instantaneous phase of the LFP can act as an additional channel operating in parallel to the usual firing-rate code and boost the amount of encoded visual and acoustic stimuli, respectively. Thus, the LFP can contain information that is not present in spike firing alone.

However, the precise mechanism by which downstream neurons could read out the information encoded by the LFP still remains elusive. Recent evidence suggests that bursting pyramidal neurons can lock their firing to a preferred phase range of the dominant LFP rhythm and this phase preference can change as a function of burst spike count (Samengo and Montemurro, 2010; Constantinou et al., 2015). Using this idea, computational models have proposed bursting as a mechanism to encode instantaneous features of an oscillating current into a pattern of spikes that can be transmitted to distant areas (Kepecs and Lisman, 2003; Samengo et al., 2013). In particular, models of pyramidal neurons suggested that intra-burst spike counts have the capacity to encode the slope (Kepecs et al., 2002) and phase (Samengo and Montemurro, 2010) of time-varying input signals.

The main hypothesis in our study is that firing single spikes and bursts of different counts can be a feasible mechanism to transmit information about local field oscillations, thus translating information in the LFP into an easily decodable code. We tested this hypothesis by a two-fold approach involving simulations from a two-compartment model of a pyramidal bursting neuron and *in-vivo* data from anesthetized and behaving rats. The model was constructed to fire with the statistics of experimentally recorded neurons and used to quantify the information about features of LFP-like oscillations in their bursting rate and intra-burst spike count. We investigated the encoding of delta and theta-dominated signals, representing LFPs of anesthetized and behaving animals, respectively. The model predicted that the output of bursting cells can indeed encode information about the instantaneous voltage, phase, slope and, to a lesser extent, amplitude of the dominant rhythms. Furthermore, there was an encoding advantage in a neural code in which single spikes, two-spike bursts and larger bursts are considered as distinct symbols compared to a code in which all these events are indistinguishable. We then tested whether the same result appeared in experimental data that we had access to: from the subiculum of anesthetized rats and the MEC of an awake behaving rat. The corresponding LFPs were dominated by delta and theta bands, respectively. The analysis, hence, allowed us to determine whether the encoding of LFP features was restricted to a specific behavioral state or frequency band, or whether it appeared as a robust mechanism in the temporal lobe. We found that a large fraction of bursting cells in both regions encoded information about LFP features in their bursting rate. In addition, some of these bursting cells also encoded information in burst size according to the model predictions. Our results suggest that LFP features can be encoded in single-cell bursting activity in the hippocampal formation of both awake and anesthetized animals.

## 2. Materials and methods

### 2.1. *In vivo* electrophysiology under anesthesia

All experiments under anesthesia were performed in accordance with the Animals (Scientific Procedures) Act UK 1986 and were approved by the University of Manchester Ethical Review Panel. Three adult male Sprague Dawley rats and one adult male Wistar rat were used. The experimental procedures for recording from the subiculum have been described before in Constantinou et al. (2015). The rats were anesthetized by intraperitoneal injection of 1.5 g/kg urethane. Their heads were fixed in a stereotaxic frame, a midline incision was made and craniotomies were drilled according to the Paxinos and Watson (2007) rat brain atlas coordinate system for subiculum (Bregma: −8.0 mm and ML: 3.5 mm). Small electrolytic lesions created at the end of the experiment indicated electrode position in Nissl-stained brain sections.

A 4 × 8 multi-electrode array was inserted at a 30° compound angle from the vertical axis to align the main axis of the electrode array parallel to the main pyramidal cell axis in the subiculum. The electrode array was attached to an electrode board and headstage and to an AC preamplifier resulting in total gain of × 2000. Simultaneous recordings of spontaneous LFP (lowpass-filtered up to 250 Hz) and spikes (highpass-filtered above 300 Hz) were obtained for an hour. Spikes were detected by setting a threshold manually for each electrode to account for differences in signal amplitude. Discrete spike shapes of 1.3 ms duration and continuous LFP (sampling rates: 40 and 2 kHz, respectively) were stored for offline analysis.

### 2.2. *In vivo* electrophysiology during awake behavior

The data from the MEC during awake behavior were recorded in a previous study (Kropff et al., 2015). All experimental procedures for the awake recordings were performed in accordance with the Norwegian Animal Welfare Act and the European Convention for the Protection of Vertebrate Animals used for Experimental and Other Scientific Purposes. A Long Evans rat was used. The rat was implanted at 3 months and recorded until 9 months.

The experimental procedures for recording from the MEC have been described before in Kropff et al. (2015). The rat was trained to run freely in a 1-m wide square box. The trials lasted at least 20 min and as long as the rat would exhibit active foraging. Tetrodes were constructed from four twisted polyimide-coated platinum-iridium wires and mounted in a group of four into a microdrive. Once the animal was anesthetized, holes were drilled on the dorsal skull anterior to transverse sinus to reach the entorhinal cortex. The coordinates for implants were: 4.5–4.8 mm medio-lateral relative to lambda, 0.7 mm anterior to the border of the sinus and 1.8 mm dorso-ventral relative to the surface of the brain. The rat was connected to the recording equipment via AC-coupled unity-gain operational amplifiers close to its head. To search for new cells, tetrodes were lowered in steps of 50 μm. The cells reported here belong to layers III and V. The LFP (lowpass-filtered up to 500 Hz, sampled at 4800 Hz) was recorded single-ended from one electrode per drive.

### 2.3. Bursting neuron model

Bursting activity was simulated using a two-compartment conductance-based model of a pyramidal neuron which has been used in previous studies (Kamondi et al., 1998; Kepecs et al., 2002; Kepecs and Lisman, 2003; Samengo and Montemurro, 2010; Constantinou et al., 2015). The model contains the minimal ionic conductances required to generate bursting activity (Kepecs and Wang, 2000) after being reduced from a 19-compartment model of a CA3 hippocampal neuron (Traub et al., 1991) to a two-compartment conductance-based model (Pinsky and Rinzel, 1994). The input current *I*(*t*) was injected into a dendritic compartment (Supplementary Equation 1) and bursting activity was recorded from a somatic compartment (Supplementary Equation 2). We had previously adjusted the model parameters (Constantinou et al., 2015) so as to produce single spikes and bursts with the same probability as subicular neurons (**Figures 2A,C**). Burst production by entorhinal neurons was governed by a similar distribution, so we only modified the variance of the input current to adapt the model to entorhinal bursting neurons (**Figures 2B,D**). The parameters and equations of the model are listed in the Supplementary Methods and Supplementary Tables 1, 2.

**Figure 1:**
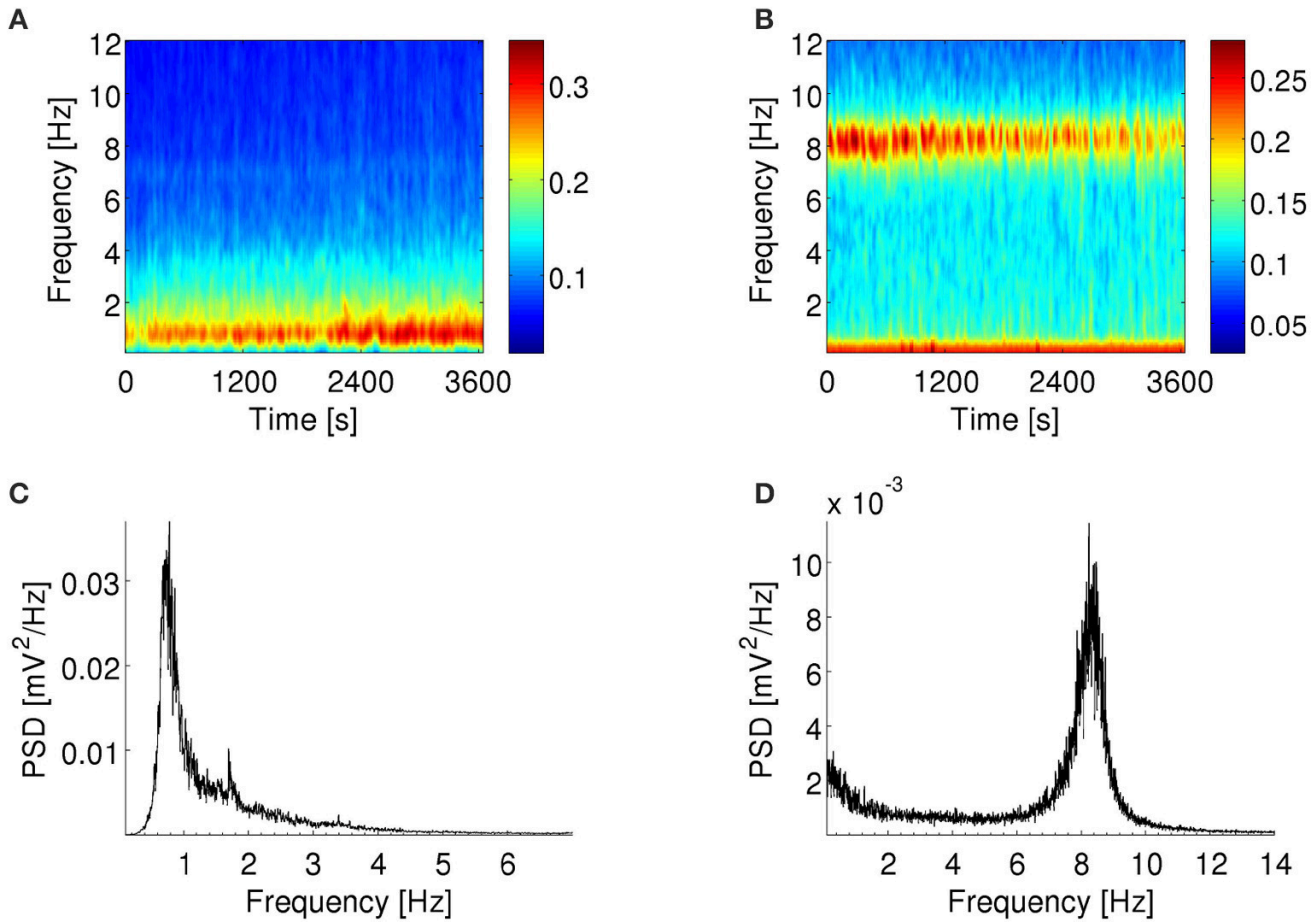
**Spectral content of LFP**. Example of spectrograms **(A,B)** and power spectra **(C,D)** of LFP recorded by an electrode in the subiculum of an anesthetized rat **(A,C)** and the MEC of an awake behaving rat **(B,D)**. **(A,C)**: LFP show a peak in spectral power at ~1 Hz throughout the recording session. **(B,D)**: LFP show a peak in spectral power at ~8 Hz throughout the recording session. There is also a smaller peak at frequencies <1 Hz. **(A,B)**: Color scale in (mV^2^/Hz)^0.25^. Warmer colors indicate higher power spectral density.

The model was used to predict the spiking activity of subicular and entorhinal neurons when immersed in oscillations present in the LFP *in vivo*. We simulated the effect of these oscillations by injecting an input current *I*(*t*), which had the same spectral structure as the experimental LFP, into the dendritic compartment of the simulated neuron. Since the LFP recordings had limited duration (1 h for subiculum and 30 min for MEC), we used a method of creating surrogate data that preserves the spectral content of LFP observed *in vivo* and can produce input signals of any desired length from a segment of LFP. To construct the input signals, a 30-min segment of the experimental LFP signal was interpolated to obtain a sampling frequency of 100 kHz and then used to create surrogate oscillatory current signals. The surrogate signals were created from the recorded trace by randomizing the phases of Fourier components and then transforming back to the time representation. Hence, the power spectra of the surrogate signals (Supplementary Figures 3A,B) are the same as their real counterpart (Figures 1C,D), but the temporal structure is altered (Theiler et al., 1992). The signals were scaled so that the mean was 0 nA and the standard deviation was 0.7 nA or 0.4 nA depending on whether the simulation corresponded to anesthetized or behaving experiments, respectively.

### 2.4. Spike sorting

For the dataset from the subiculum, the spike shapes recorded from each electrode were imported in Offline Sorter V2.8.8 (Plexon Inc.) to isolate spikes from individual neurons. Different combinations of spike shape parameters were chosen for clustering until units were identified and manually separated. Units that were difficult to isolate from the background noise were discarded. The quality of separation was assessed by visual inspection of interspike interval (ISI) histograms to ensure no spikes were present within the neuronal refractory period of 1 ms. To identify multiple detection of the same unit on adjacent electrodes, cross-correlograms were plotted for each unit vs. all the other units. For pairs of units with apparent cross-correlation, indicated by a large peak within 1 ms from zero, only the unit with the largest spike waveforms was used for subsequent analyses.

For the dataset from the MEC, spikes were assigned to individual neurons offline using the graphical cluster-cutting software TINT (Axona Ltd.), as described in Kropff et al. (2015). The procedure was analogous to that for the dataset from the subiculum.

### 2.5. Identification of bursting neurons and spike train segmentation

Bursting units were identified from ISI histograms and autocorrelograms of spike times recorded at each electrode. Units in the subiculum were classified as bursting if the ISI histogram and the autocorrelogram had a sharp peak within 2–8 ms and these peaks were larger than any other peak within 50 ms (Supplementary Figures 1A,B). Units in the MEC were classified as bursting if the sharp peak was within 2–5 ms (Supplementary Figures 1C,D). These criteria are consistent with previous studies characterizing bursting units as having a peak within 6 or 10 ms (Ranck, 1973; Harris et al., 2001; Mizuseki et al., 2009).

Consecutive spikes separated by less than 8 or 5 ms (in subiculum and MEC, respectively) were assigned to the same burst. These thresholds were larger than the prominent peak in the ISI histograms (Supplementary Figures 1A,C). Changing the 8 ms threshold to 6 or 10 ms gave qualitatively similar burst size distributions, phase locking and information patterns (data not shown) so spike segregation in bursts was robust to small differences of threshold.

The time-scale of the response patterns of the simulated neurons was slower, since the prominent peak of the ISI distribution appeared at longer times (Supplementary Figures 3C–F). Hence, consecutive spikes were assigned to the same burst when the ISI was below 16 ms.

### 2.6. Spectral analysis and data segmentation

LFP and input signals to the model were resampled to 200 Hz to reduce computation time. Decimation was used in order to prevent the aliasing effect of signal components above the Nyquist frequency in the downsampled signal. To visualize the spectral content of LFP signals, power spectra were plotted using the Welch's periodogram method with Hamming windows of 200 s and 50% overlap (Figures 1C,D). To depict how the power of LFP oscillations changed over the duration of the experiment, the Fourier decomposition of the signal across time and frequency was visualized in spectrograms computed with Hamming windows of 2 s and 50% overlap (Figures 1A,B). For illustration purposes in Figures 1A,B and Supplementary Figure 2A only, the spectrograms were smoothed with a 200-ms moving window to overcome excessive pixelation of the image.

In each rat, the power spectra of the LFP recorded from all electrodes in the subiculum or MEC were remarkably similar. During the 1-h recording under urethane-anesthesia, there was a prevalent peak at ~1 Hz (example in Figures 1A,C) and for three of the four rats there were epochs in which the network shifted transiently to a different dynamical state, dominated by a peak at ~3–4.5 Hz (example in Supplementary Figure 2). The first peak corresponded to delta rhythms and the latter to theta rhythms as recorded under urethane anesthesia. The ~1 Hz rhythm under similar experimental conditions has also been referred to as hippocampal slow oscillations in the literature (Wolansky et al., 2006; Clement et al., 2008). We isolated the epochs with dominant delta rhythms as described in Constantinou et al. (2015). In summary, based on the power spectra, the frequency bands for delta and theta rhythms were defined as 0.5–2.5 Hz and 2.5–5.0 Hz, respectively. Small changes in the boundaries of these bands did not affect the results in pilot analyses. The dominant rhythm was defined as the band with the highest power at a given time point at which the difference between the power of this band and any other band was at least 10%. The epochs with dominant theta rhythms under anesthesia are discussed in the Supplementary Results and Supplementary Figures 8, 9. The LFP recordings from the awake rat during foraging activity contained a prominent spectral peak at ~8 Hz (example in Figures 1B,D). This frequency corresponds to the theta rhythm associated with exploratory behavior and was stable throughout the recordings.

### 2.7. LFP filtering and feature extraction

LFPs were filtered using a finite impulse response (FIR) digital filter with Kaiser window (sharp transition bandwidth: 1.0 Hz, stopband attenuation: 60 dB, passband ripple: 0.01 dB). LFPs were bandpass-filtered with cut-off frequencies 0.5 and 3 Hz to extract the delta rhythm in the anesthetized data, or 6 and 12 Hz to extract the theta rhythm in the awake data. For the systematic narrowband analysis of Figures **8–11** and Supplementary Figures 5–7, 9, the LFP signals were filtered in 1 Hz windows with 75% overlap, except for the first frequency window which ranged from 0.1 to 1 Hz.

Features were extracted from the filtered LFP signals. The investigated features were the instantaneous voltage (or input signal for the model), slope, phase, and amplitude. Slope was calculated as the derivative of the LFP (experiments) or input signal (simulations). Phase and amplitude were computed as the argument and modulus, respectively, of the complex Hilbert transform of the LFP or input signal. With our angular convention, a phase of 0° corresponded to a maximum in the oscillatory signal.

### 2.8. Information measures

Information theory (Shannon, 1948) was used to quantify how much information about LFP features can be conveyed by the output of bursting neurons. In the case of simulated neurons, the features of the LFP are replaced by the same features of the input current *I*(*t*) injected into the model. Information was defined as the average reduction in uncertainty about a given LFP feature by knowing the neuronal output.

To estimate information measures, time was binned into small intervals of duration δ*t* = 5 ms. Each interval was associated with a neural response and a LFP feature. The latter could be either synchronous with the neural response (no time lag) or could be located at a fixed time before or after the response. The collection of all the values of a given feature throughout a session defined the feature set *X*.

**Figure 2:**
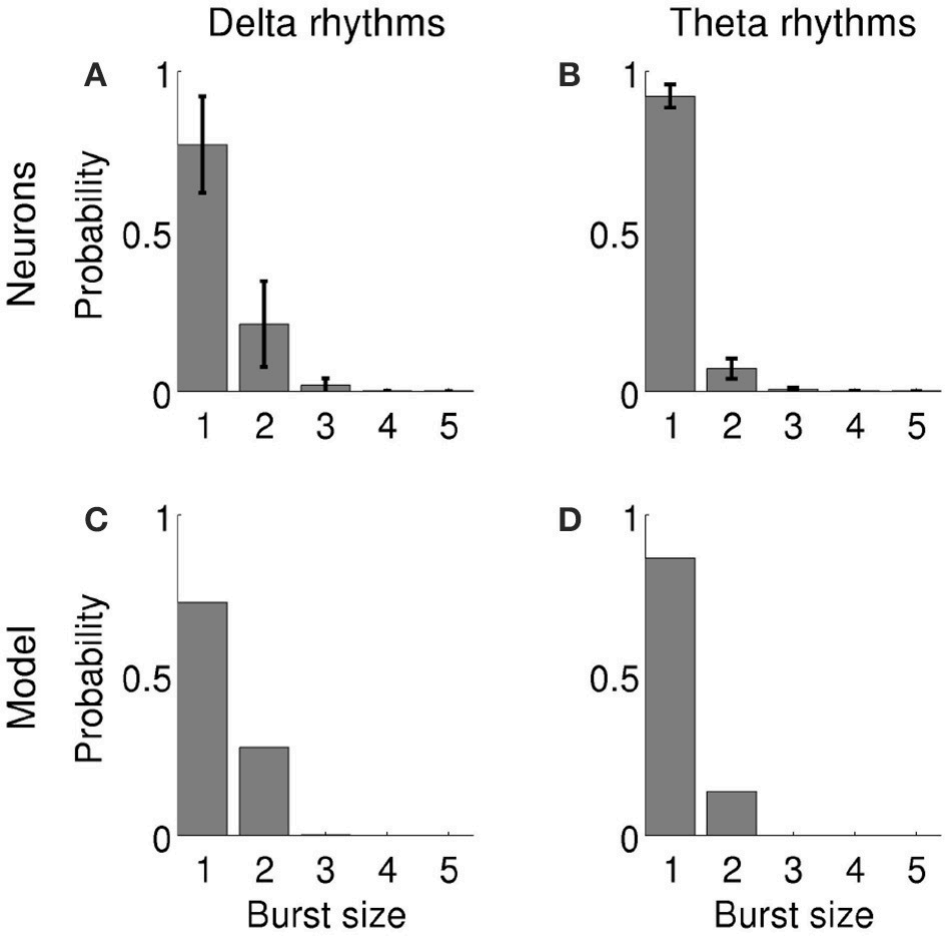
**Probability of firing *n*-spike events by bursting neurons in the subiculum when delta rhythms were dominant in the LFP under anesthesia (A)** and in the MEC when theta rhythms were dominant in the LFP during awake behavior **(B)**. Bars show the average probability across 28 units in subiculum **(A)** and 42 units in MEC **(B)**; error bars indicate standard deviation. **(C,D)**: Probability of the model firing *n*-spike bursts when delta **(C)** or theta rhythms **(D)** were dominant in the input signal.

We studied three possible ways—referred to as *full burst code, burst rate code* and *burst distinction code*—by which bursting neurons encode LFP features. For the full burst code, the set *N* of all possible neuronal responses consisted of four distinct symbols: no spike (*n* = 0), single spike (*n* = 1), two-spike burst (*n* = 2) and larger burst (*n* = 3). Bursts of three or more spikes were represented by the same symbol because they occurred rarely (Figure 2). Each time bin was associated with one such response, located at the time of burst initiation. The burst rate code was obtained from the full burst code by considering all bursts containing one or more spikes (*n* ≥ 1) as indistinguishable events. Hence, the 0s of the full burst code were preserved in the burst rate code and a new symbol representing the initiation of a burst replaced all other *n* values. The burst distinction code differed from the previous two in that only a subset of the time bins was employed: the time bins where a burst was initiated. That is, all the time bins associated with a 0 response were discarded. Neuronal activity was described by a response set *N* = {1, 2, 3} which distinguished between bursts of different spike count. The information encoded by the burst distinction code quantifies whether bursts of different sizes are useful to discriminate LFP features. The data processing inequality (Cover and Thomas, 2006) ensures that the full burst code cannot encode less information than any of the other codes and equality is only possible if the discarded aspect is irrelevant to information encoding.

When the time bin is sufficiently brief, the information *I*(*X*; *N*) about a LFP feature (*X*) conveyed by bursts (*N*) with the full burst code or with the burst rate code can be estimated by adapting the method described in Skaggs et al. (1993), to incorporate the firing rate of *n*-spike bursts (Eyherabide et al., 2008) so that:

(1) $$I(X;\text{​}N)=\delta t\;\sum_{n\in N}\sum_{x\in X}p(x)r_{n}(x)\log_{2}\frac{r_{n}(x)}{r_{n}},$$

where *p*(*x*) is the probability of each LFP feature value and *r*_*n*_(*x*) is the rate of each *n*-spike event conditional to a LFP feature of value *x*. The average rate of each *n*-spike event *r*_*n*_ is:

(2) $$r_{n}=\sum_{x\in X}p(x)r_{n}(x).$$

The information values obtained from Equation (1) are in units of bits per time bin. The information was converted to bits/burst by dividing the value obtained from Equation (1) by the average number of bursts in a time bin, that is, by δ*t r*, where *r* is the total burst rate.

In the full burst code: *N* = {0, 1, 2, 3}, in the burst rate code: *N* = {0, burst}, and in the burst distinction code: *N* = {1, 2, 3}. Applying the chain rule *I*(*X*; *Y, Z*) = *I*(*X*; *Y*) + *I*(*X*; *Z*|*Y*) to the case *Y* = {0, burst}, *Z* = {1, 2, 3}, the three codes are related by *I*(*X*; {0, 1, 2, 3}) = *I*(*X*; {0, burst}) + *rδtI*(*X*; {1, 2, 3}) (derivation in Supplementary Methods). Therefore, in order to calculate the information per burst encoded in the burst distinction code, one may calculate the difference:

(3) $$I(X;\text{​}\{1,2,3\})=\frac{1}{\delta t\;r}\;\left[I(X;\text{​}\{0,1,2,3\})-I(X;\text{​}\{0,\text{burst}\})\right].$$

Alternatively, the information of the burst distinction code can be computed directly from the Shannon equation *I*(*X*; *N*) = *H*(*X*) − *H*(*X*|*N*) with *N* = {1, 2, 3} and defining *X* as the set of features associated with the time bins where a burst was fired.

The continuous values of the LFP features were discretized into four symbols to define the set *X* (a justification of the chosen binning is given in the Supplementary Methods and Supplementary Figure 4). The boundary of bins was adjusted such that the distribution of the four symbols was uniform. Hence, the probability of each symbol *x* was *p*(*x*) = 0.25.

Due to the finite nature of experimental data, the estimated probabilities used to compute mutual information contain statistical errors, which lead to a sampling bias in the information estimators. The bias is defined as the difference in the information values calculated from the probabilities estimated from experimental data and from the true probabilities (Panzeri et al., 2007). To correct for this bias, a bootstrapping method (Montemurro et al., 2007a,b, 2008) was used. The burst size labels corresponding to each LFP feature value were shuffled and the mutual information *I*_*s*_(*X*; *N*) was calculated with the shuffled data. Although in principle shuffling eliminates all statistical correspondence between burst size and LFP features, the resulting information value still does not vanish, due to the bias. The procedure was repeated 100 times, and the average of the shuffled information values 〈*I*_*s*_(*X*; *N*)〉 was taken as an estimation of the sampling bias. Since the output statistics varied across cells, the bias estimation was done individually for each cell.

A given cell was considered to convey a significant amount of information about a given feature when the information obtained with the real data was larger than the maximum value of the 100 shuffled information estimates across time. This maximum value could happen at any point in the time window around burst onset. For significantly encoding cells, the bias-corrected information *I*_*c*_(*X*; *N*) was obtained by subtracting 〈*I*_*s*_(*X*; *N*)〉 from the mutual information estimate *I*(*X*; *N*). The bias-corrected information is hereafter referred to as information.

### 2.9. Phase-locking estimation

For each cell and *n* value, phase-locking was estimated by calculating the probability of firing a burst of *n* spikes conditional to a LFP phase of a specific range. The interval [−180^*o*^, 180^*o*^] was divided into 25 phase ranges, each of size 14.4°. The phase was computed at the time of burst onset.

### 2.10. Principal component analysis

In order to determine whether pairwise correlations suffice to explain all the structure in the statistics of the information data, we performed a principal component analysis (PCA) of the information transmitted about the four features at the population level. Each cell in either subiculum or MEC was taken as a sample of a 4-dimensional vector **v**_*i*_, whose components were the values of the mutual information obtained with the full burst code about the four explored LFP features (voltage, slope, phase and amplitude). The 4 × 4 covariance matrix of each population (subiculum or MEC) is:

(4) $$C=\bar{\left(v_{i}-\bar{v_{i}}\right)\;{\left(v_{i}-\bar{v_{i}}\right)}^{T}},$$

where the horizontal bar represents a population average on all the bursting cells *i* of each brain area, and the supra-script *T* stands for vector transposition. The eigenvectors of *C* are orthogonal, and indicate the directions in which information vectors are uncorrelated. The associated eigenvalues are always non-negative and equal to the variance of the population data along the direction of the corresponding eigenvector. If one of the eigenvalues is much larger than the other three, then the information about the different features is strongly correlated throughout the population and all information vectors are essentially proportional to the principal eigenvector (the one associated with the largest eigenvalue). The eigenvector associated to the second eigenvalue indicates an additional direction of variability which, although less important, implies fluctuations in information values that are uncorrelated with those in the principal direction.

## 3. Results

We investigated how bursting neurons encode information about LFP features in the hippocampal formation using both a bursting neuron model and electrophysiological data recorded *in-vivo* from the subiculum and the MEC. Three possible ways of transmitting information were explored: the full burst code, burst rate code and burst distinction code (see Materials and Methods). Each code corresponds to a different representation of the bursting responses. The full burst code considers both the timing and the spike count of each burst, representing the *when* and *what* of the encoded features, respectively (Eyherabide and Samengo, 2010a,b). In the burst rate code, only the timing of bursts is represented; and in the burst distinction code, only the spike count.

For shortage of notation, we employ the word *burst* to all spike patterns including not only sequences of two or more spikes, but also single spikes, which are considered as one-spike bursts. In all cases, the statistical correspondence between LFPs and bursting responses was explored using the LFP recorded at the same electrode where the single-cell activity was registered.

### 3.1. Information encoded by simulated bursting neurons

In order to mimic the effect of the fluctuating extracellular medium on neuronal excitability, we used variations of the LFP recorded in the experimental data as the input signal driving a simulated neuron (see Methods for input signal construction and computational model). The LFPs recorded in anesthetized and behaving animals contained markedly different spectral characteristics (Figure 1). Therefore, each of these conditions was simulated independently using a driving signal with the corresponding spectral profile (Supplementary Figure 3). The firing statistics of the simulated neuron were similar to the *in-vivo* recorded cells (Figure 2).

**Figure 3:**
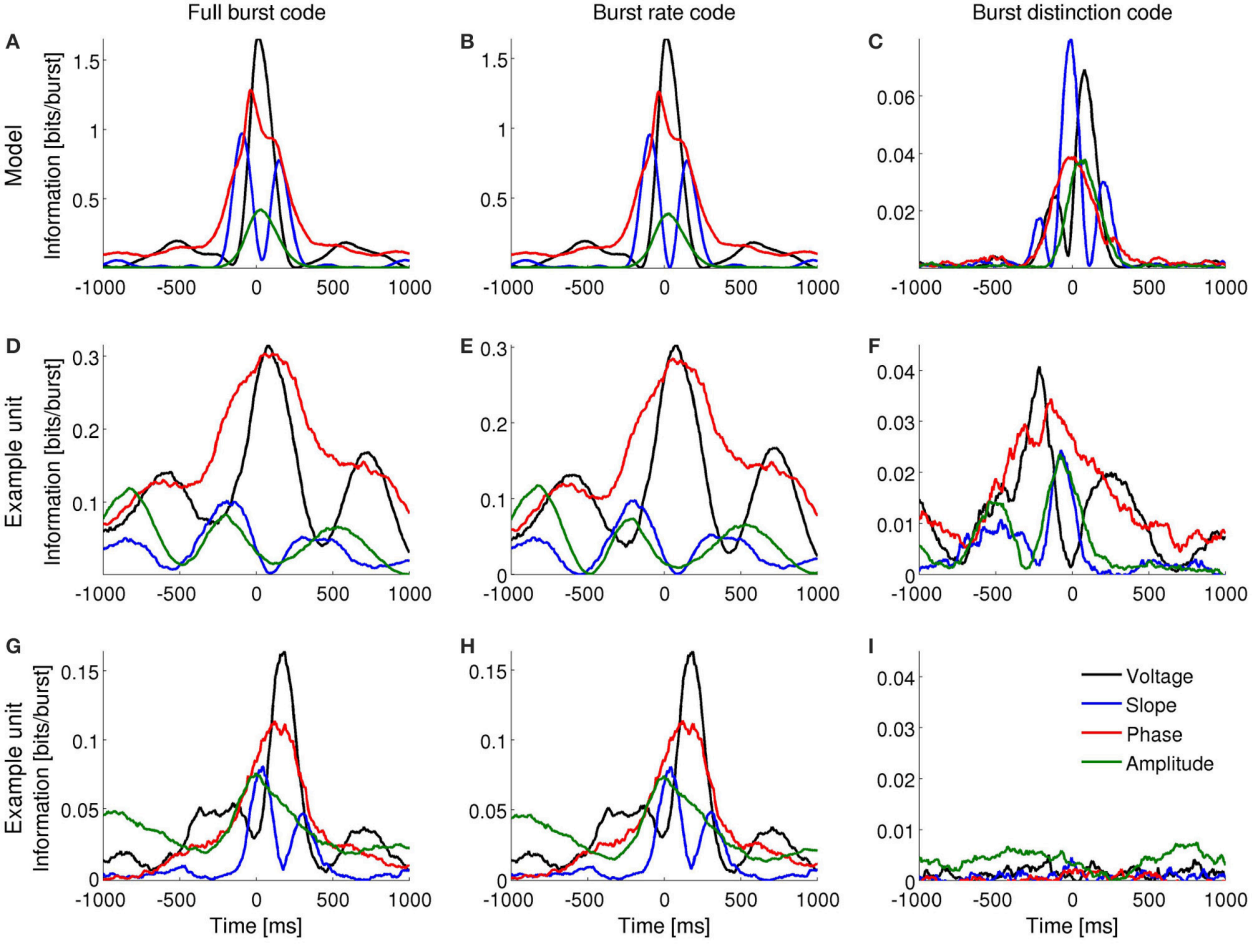
**Information encoded by bursting neurons about the instantaneous voltage, slope, phase and amplitude of the delta-filtered LFP. (A–C):** Mutual information obtained with the computational model when the input signal contains dominant delta rhythms. **(D–F)** and **(G–I)**: Mutual information obtained for two different subicular cells under anesthesia. Both cells encode information about the voltage, slope, phase and amplitude of delta-filtered LFP by the full burst code **(D,G)** and burst rate code **(E,H)**. One of the cells encodes information about LFP features in the distinction between different burst sizes **(F)** whereas the second does not **(I)**.

**Figure 4:**
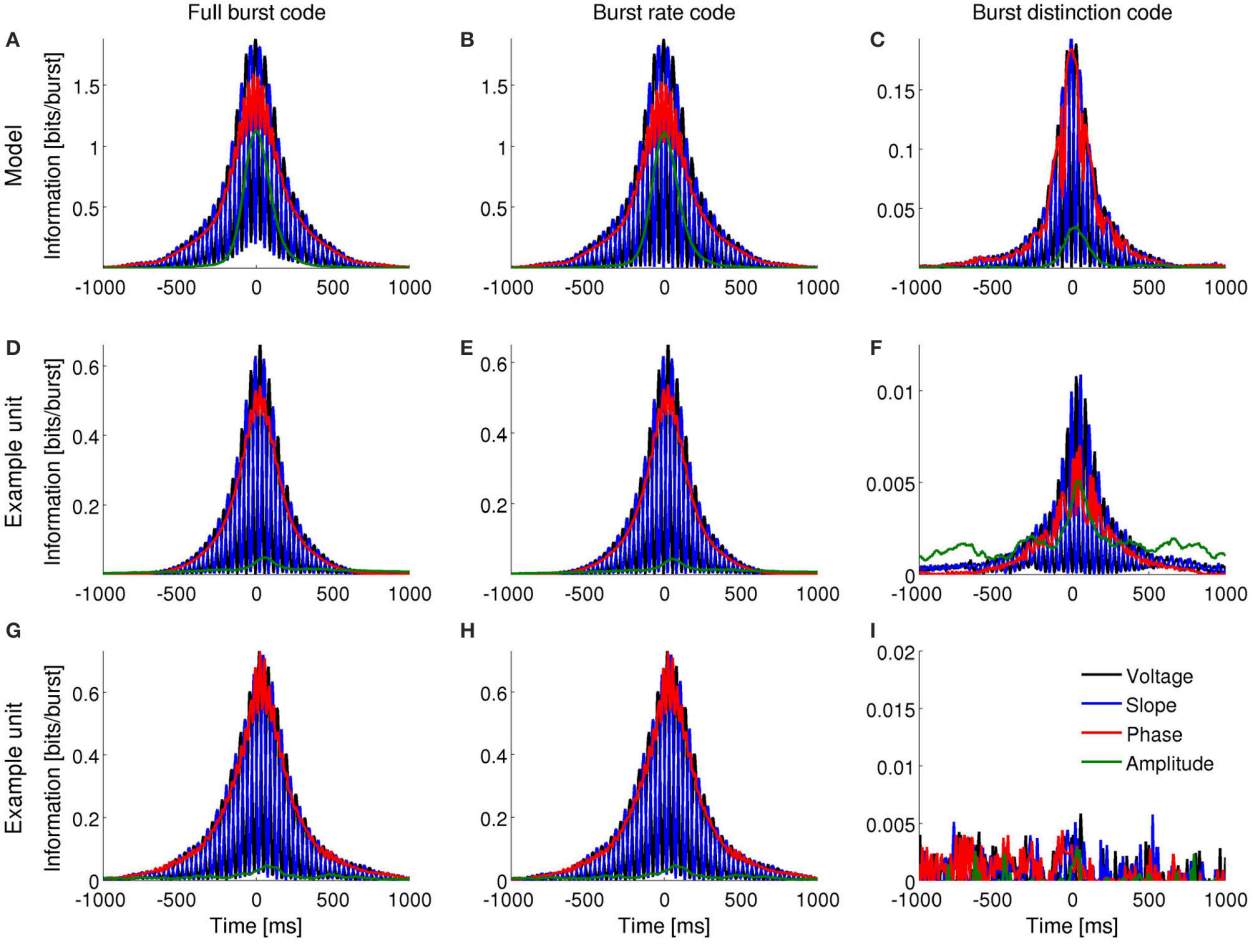
**Information encoded by bursting neurons about the instantaneous voltage, slope, phase and amplitude of the theta-filtered LFP**. **(A–C)**: Mutual information obtained with the computational model when the input signal contains dominant theta rhythms. **(D–F)** and **(G–I)**: Mutual information obtained for two different entorhinal cells during foraging behavior. Both cells encode information about the voltage, slope and phase of theta-filtered LFP by the full burst code **(D,G)** and burst rate code **(E,H)**. One of the cells encodes information about LFP features in the distinction between different burst sizes **(F)** whereas the second does not **(I)**.

Neurons integrate information over time and, at a certain moment, fire a response (or not). Therefore, responses are not only sensitive to the instantaneous properties of the input signal, they also depend on its past history. Moreover, if the signal contains temporal correlations, the past values of the signal are correlated with its future values. Hence, a given event in the neural response may predict a future signal feature. Indeed, neuronal bursting was not only modulated by features occurring at the time of burst initiation, but also, to a lesser extent, by features appearing up to 200 ms before or after (Figures 3A–C, 4A–C). Out of the four tested features (instantaneous value of the input signal *I*(*t*) and the associated slope, phase and amplitude), the best encoded features were the instantaneous value, phase and slope. The information about *I*(*t*) and slope oscillated with a frequency that doubled the frequency of the dominant rhythm, both for delta and theta-dominated inputs (Figures 3A–C, 4A–C). This effect is explained at the end of Section 3.4.

A full burst code, in which all *n*-spike bursts—where *n* indicates the intra-burst spike count and *n* = 0 for time bins where there is no event fired—corresponding to each instantaneous LFP feature are distinct symbols, encoded slightly more information than a burst rate code, in which the size of bursts was indistinguishable (Figures 3A,B, 4A,B). The information obtained with the burst distinction code, which considers the spike count *n* only in the time bins where a burst was registered, was approximately 10–20 times smaller than with the other two codes (Figures 3C, 4C). These results imply that most of the encoded information was temporal. In other words, the simulated neuron mainly detected *when* a given feature fell within a specific range and, to a lesser extent, encoded finer distinctions in the intra-burst spike count.

Bursting neurons *in-vivo* exhibited a range of patterns of information encoding, often resembling the simulated neuron. Figures 3D–I, 4D–I show examples in the subiculum under anesthesia and the MEC during awake behavior, respectively.

### 3.2. Population analysis of subicular neurons

**Figure 5:**
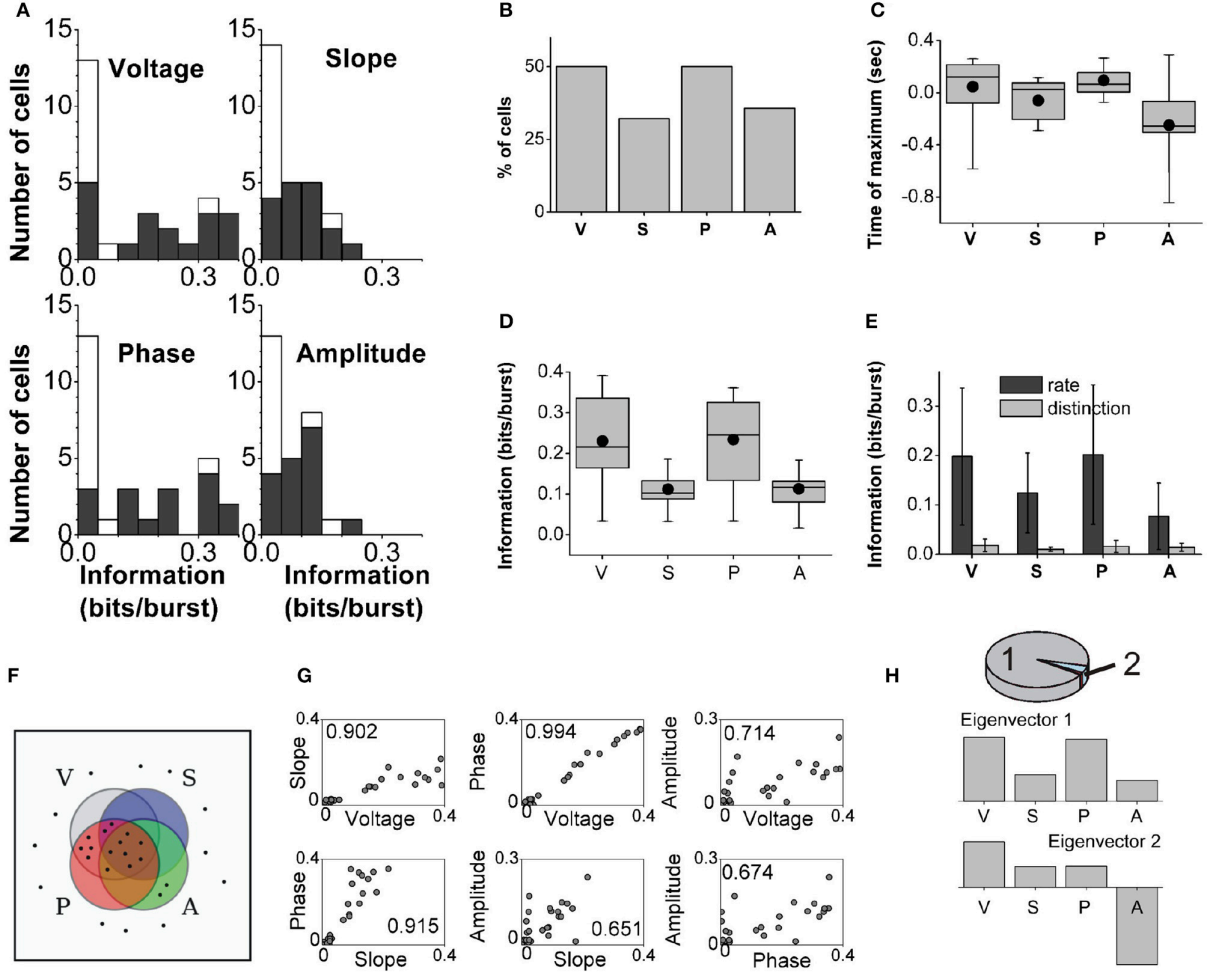
**Population analysis of the information encoded by subicular bursting neurons about the delta-filtered LFP**. **(A)**: Histograms displaying the information encoded by different cells in the population about the four explored features. Black and white areas represent cells with significant and non-significant amounts of information, respectively. **(B)**: Fraction of cells encoding significant information of at least 0.1 bits/burst. **(C)**: Population statistics of the time relative to burst onset at which the information encoded by the full burst code reached its maximum value (only significant values included). Black dot: mean; horizontal bar: median; upper and lower borders of the box: 25th and 75th percentiles; thin lines: maximum and minimum values. **(D)**: Population statistics of the maximal information encoded by the full burst code. Box representation same as in **(C)** (significant values only). **(E)**: Comparison of the mean maximal information encoded by the burst rate and burst distinction codes (significant values only). Error bars report standard deviation. **(F)**: Schematic representation of which features are encoded by each cell in the population. Each cell is indicated as a dot, and each set encloses only the cells that encoded at least 0.1 bits/burst about voltage (V), slope (S), phase (P) or amplitude (A). **(G)**: Pearson correlation coefficients between the maximal information encoded by each bursting neuron (shown as a dot) about all pairs of features. **(H)**: Principal component analysis (PCA) in which each cell is taken as a sample vector, and each feature as a dimension. Top: 98% of the variance is explained by only two eigenvectors, 94 and 4% respectively. Middle and bottom: First two eigenvectors obtained by PCA.

We identified 28 bursting units in the subiculum of anesthetized rats during states with predominant delta rhythms. The probability of firing *n*-spike bursts decreased with the intra-burst spike count (Figure 2A). The population distributions of information values obtained with the full burst code are displayed in Figure 5A. For each cell in the population and each feature, the reported information values correspond to features evaluated at the time where information was maximal. The dark bars show significant information values, and the light bars show non-significant information values (information values below threshold). There were cells that encoded up to 0.4 bits/burst about voltage and phase, whereas the values corresponding to slope and amplitude were typically lower. The fraction of cells encoding significant information of at least 0.1 bits/burst about the voltage, slope, phase and amplitude were 50.0, 32.1, 50.0, and 35.7%, respectively (Figure 5B).

The information encoded by subicular cells about each LFP feature reached its maximum value for features occurring synchronously, before or after burst onset (examples in Figure 3). The distributions of times at which information was maximized with the full burst code are summarized in Figure 5C. Most subicular neurons encoded maximal information about features occurring approximately 200–300 ms before or after burst onset. At the population level, the timing of maximal information about voltage and amplitude swept a wider range than for slope and phase. For 81.3% of the cells encoding significant information about phase, the timing of the maximal information corresponded to future phase values. For 82.4% of the cells encoding significant information about amplitude, the timing of the maximal information corresponded to past amplitude values. Therefore, bursting neurons can encode information about both past and future features of the delta-filtered LFP.

The distributions of significant information values for the full burst code are summarized in Figure 5D. Some of the four distributions had significantly different medians (Kruskal-Wallis test: χ^2^ = 18.57, *df* = 67, *p* = 0.0003; followed by Tukey-Kramer multiple comparisons test of the averaged group ranks). In particular, at the population level, the median information about voltage and phase was not significantly different, nor was the information about slope and amplitude. However, the median information about voltage was significantly different from slope and amplitude, and also the median information about phase was significantly different from slope and amplitude.

The comparison between the population averages of the information encoded in the burst rate and burst distinction codes is summarized in Figure 5E. The population averages of the ratio *I*_distinction_/*I*_full_ were 13.8, 12.6, 13.1, and 21.2% for voltage, slope, phase, and amplitude, respectively. This indicates that most information was encoded in the timing of bursts and a smaller fraction in the distinction between burst sizes.

Figure 5F depicts the population profile of feature representation. Each neuron is indicated as a dot, and the set of each feature includes the neurons that encoded significant information of at least 0.1 bits/burst. More than half of the cells (57.1%) encoded at least one of the four features and thus appear inside of at least one of the sets. Out of all cells, 25.0% encoded all four features and thus appear in the intersection of the four sets; 14.3% encoded only voltage and phase; 7.1% encoded voltage, slope and phase but not amplitude; 7.1% encoded only amplitude; and 3.6% encoded voltage, phase and amplitude but not slope.

Figure 5G shows that the information about the four LFP features was typically pairwise correlated, most notably between phase and voltage. Amplitude was the most independently encoded feature. PCA indicated that most of the variance (94%, Figure 5H, top) in the distribution of information values was captured by an eigenvector whose predominant components included voltage and phase, and to a minor extent, slope and amplitude (Figure 5H, middle). An additional 4% of the variance was captured by a second eigenvector that had a large component in the direction of amplitude (Figure 5H, bottom). These results underscore that a large fraction of cells encoded the four features simultaneously, with more information encoded about phase and voltage, and less about slope and amplitude. An independent subset of cells encoded predominantly the amplitude as indicated by the second eigenvector.

### 3.3. Population analysis of entorhinal neurons

**Figure 6:**
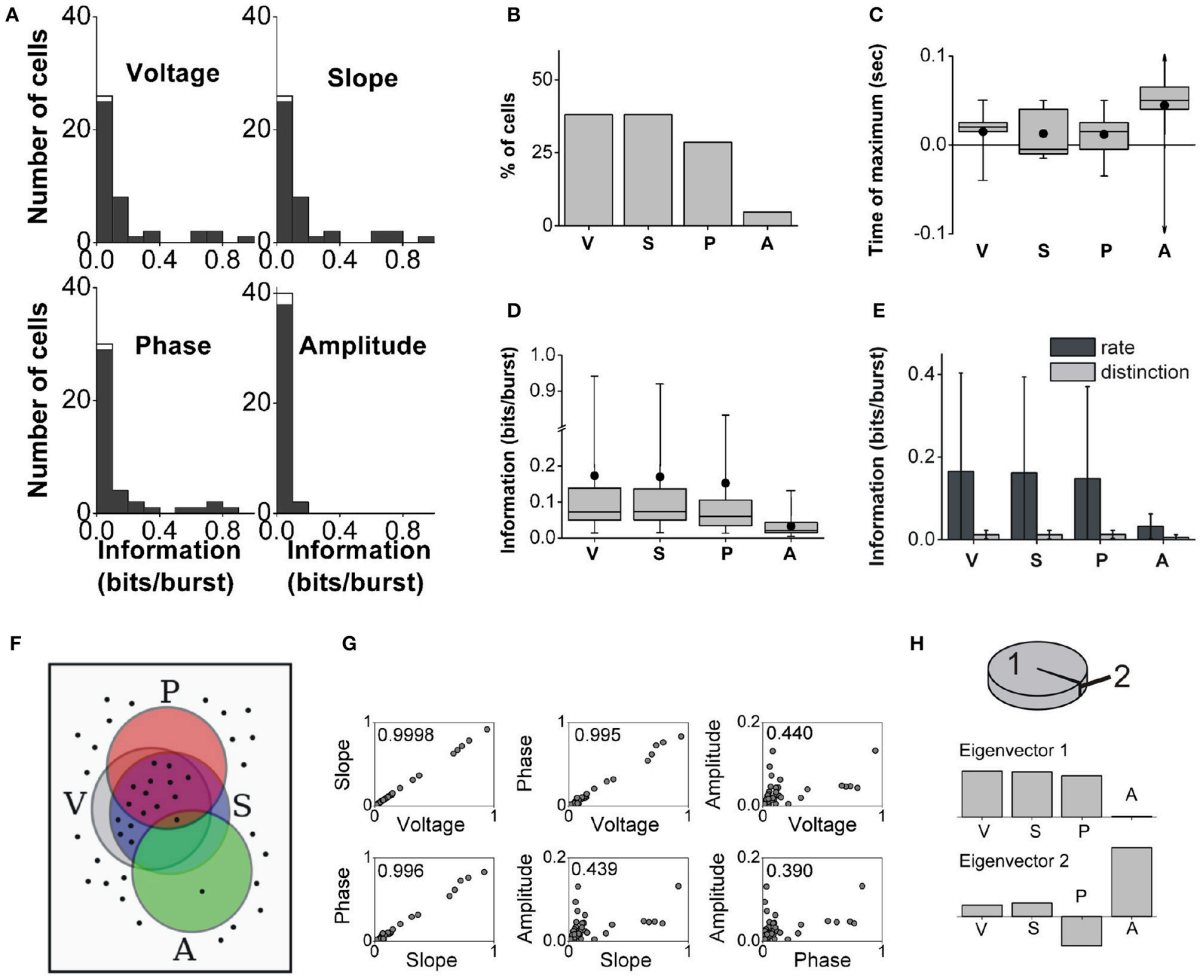
**Population analysis of the information encoded by entorhinal bursting neurons about the theta-filtered LFP**. Panels same as in Figure 5. **(C)**: The largest and smallest times of maximal information about amplitude were +780 and −880 ms (out of scale). **(D)**: Note the break in the *y*-axis. **(H)**: 99.7% of the variance is explained by only two eigenvectors: 99 and 0.7% respectively.

We identified 42 bursting units in the MEC of the awake behaving rat during theta rhythms. Burst firing probability decreased as the intra-burst spike count increased (Figure 2B). The population distributions of information values obtained with the full burst code are displayed in Figure 6A. The histograms corresponding to voltage and slope are remarkably similar, and all but amplitude contain long tails with high-information values. There were cells that encoded more than 0.9 bits/burst about voltage and slope, and more than 0.8 bits/bursts about phase. The maximal information about amplitude was notably lower (0.13 bits/burst). The fraction of cells encoding significant information of at least 0.1 bits/burst about the voltage, slope, phase and amplitude were 38.1, 38.1, 28.6, and 4.8%, respectively (Figure 6B).

Similarly to subicular neurons, the maximal information encoded by bursting cells in the MEC could correspond to features occurring synchronously, before or after burst onset (examples in Figure 4). The distributions of times of maximal information for the full burst code are summarized in Figure 6C. At the population level, entorhinal neurons encoded maximal information about the instantaneous voltage, slope and phase within 50 ms before or after burst onset; whereas maximal information about amplitude could be up to approximately 800–900 ms around burst onset. Maximal information tended to correspond to future feature values of the theta-filtered LFP, in particular, 87.8% of the encoding cells conveyed maximal information for future voltage values.

The distributions of significant information values for the full burst code are summarized in Figure 6D. The long tails obtained for voltage, slope and phase produced mean information values that were notably larger than the medians. At the population level, the median information about voltage, slope and phase was not significantly different, but the median information about amplitude was different from the other three (Kruskal-Wallis test: χ^2^ = 42.5, *df* = 161, *p* = 3 × 10^−9^; followed by Tukey-Kramer multiple comparisons test of the averaged group ranks).

The comparison between the population averages of the information encoded in the burst rate and burst distinction codes is summarized in Figure 6E. The population averages of the ratio *I*_distinction_/*I*_full_ were 16, 16, 23, and 13% for voltage, slope, phase and amplitude, respectively. Thus, the timing of bursts encoded most of the information, and intra-burst spike counts encoded a smaller fraction of the information.

Figure 6F illustrates that 40% of the entorhinal bursting cells encoded at least 0.1 bits/burst of one or more of the four features and thus appear inside at least one of the feature sets. Out of all cells, 26% encoded at least 0.1 bits/burst of information about voltage, slope and phase but not amplitude; 10% only voltage and slope; 2% only amplitude; and 2% all four features.

The information about the four different features was typically pairwise correlated, most notably, between voltage, slope, and phase (Figure 6G). Amplitude was the most independently encoded feature. The PCA indicated that 99% of the variance (Figure 6H, top) was captured by an eigenvector with predominant components along voltage, slope, and phase (Figure 6H, middle). An additional 0.7% of the variance was captured by a second eigenvector that had a large component in the direction of amplitude (Figure 6H, bottom). Hence, most cells encoded voltage, slope and phase simultaneously, and an independent subset of cells encoded a small amount of information about amplitude.

### 3.4. Burst-triggered averages

**Figure 7:**
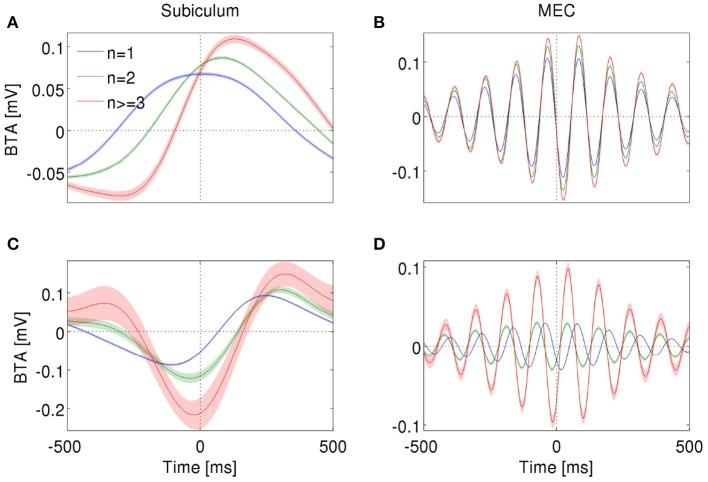
***n*-BTA of LFP around single spikes (blue), two-spike bursts (green) and larger bursts (red) fired by subicular neurons during epochs with dominant delta rhythms in the LFP (A,C)** or entorhinal neurons during dominant theta rhythms **(B,D)**. Each example is from a different bursting unit. LFP was filtered within 0.5–3 Hz **(A,C)** or 6–12 Hz **(B,D)**. Shade shows standard error of mean. Spike or burst onset is at time = 0 ms.

In order to gain insight about how bursting neurons encode LFP features, for each cell and *n* value, we calculated the *n*-burst triggered average (*n*-BTA), that is, the average bandpass-filtered LFP around *n*-spike bursts. In both subiculum and MEC, the *n*-BTA revealed that specific *n* values were predominantly associated with specific LFP features and the code varied from cell to cell. To illustrate these variations, two example units from each area are shown in Figure 7.

The subicular unit of Figure 7A fired bursts near a maximum of the LFP, whereas the one in Figure 7C fired near the trough. In both examples, the slope and amplitude of the LFP around burst initiation (*t* = 0) changed with increasing spike count *n*. Instantaneous phase changed with *n* only for the cell in Figure 7A, whereas in Figure 7C, all bursts were triggered at the minimum of the LFP, irrespective of *n*. At the time of burst onset, voltage varied with *n* in Figure 7C but not in Figure 7A. The information encoded by the cell of Figure 7A is shown in Figures 3D–F.

The entorhinal unit of Figure 7B encoded LFP features both in the burst rate (Figures 4D,E) and, to a much smaller extent, in the distinction between bursts of different spike-count (Figure 4F). Accordingly, the *n*-BTAs of Figure 7B are all similar, implying that bursts of different sizes hardly discriminate between LFP features. The cell in Figure 7D shows a different case, where the instantaneous voltage, slope, phase and amplitude vary with *n*. Hence, the distinction between bursts of different size provides information about the four features.

Figure 7 is useful to understand why the information plots in Figures 3, 4 display oscillating patterns for voltage and slope (but not for phase and amplitude) and why the frequency of the oscillations doubled the dominant frequency of the LFP. The LFP typically remains coherent during several cycles. The voltage therefore displays a rather regular oscillatory pattern. Whenever the BTAs corresponding to different *n* values cross each other, the distinction between these *n* values cannot convey information about voltage. The crossings occur at twice the dominant frequency, so this is the frequency at which information necessarily drops significantly. If all the *n*-BTAs cross simultaneously, information drops down to zero. If only some of the *n*-BTAs cross at a given time, the information decreases, but does not necessarily vanish. The same argument can be constructed for the slope of the LFP, since the slope is also an oscillatory signal and crossings occur at twice the dominant frequency. The case of instantaneous phase and amplitude is different, since they are not constrained to oscillate, and if they do, their frequency is not fixed.

### 3.5. Burst generation and phase locking

**Figure 8:**
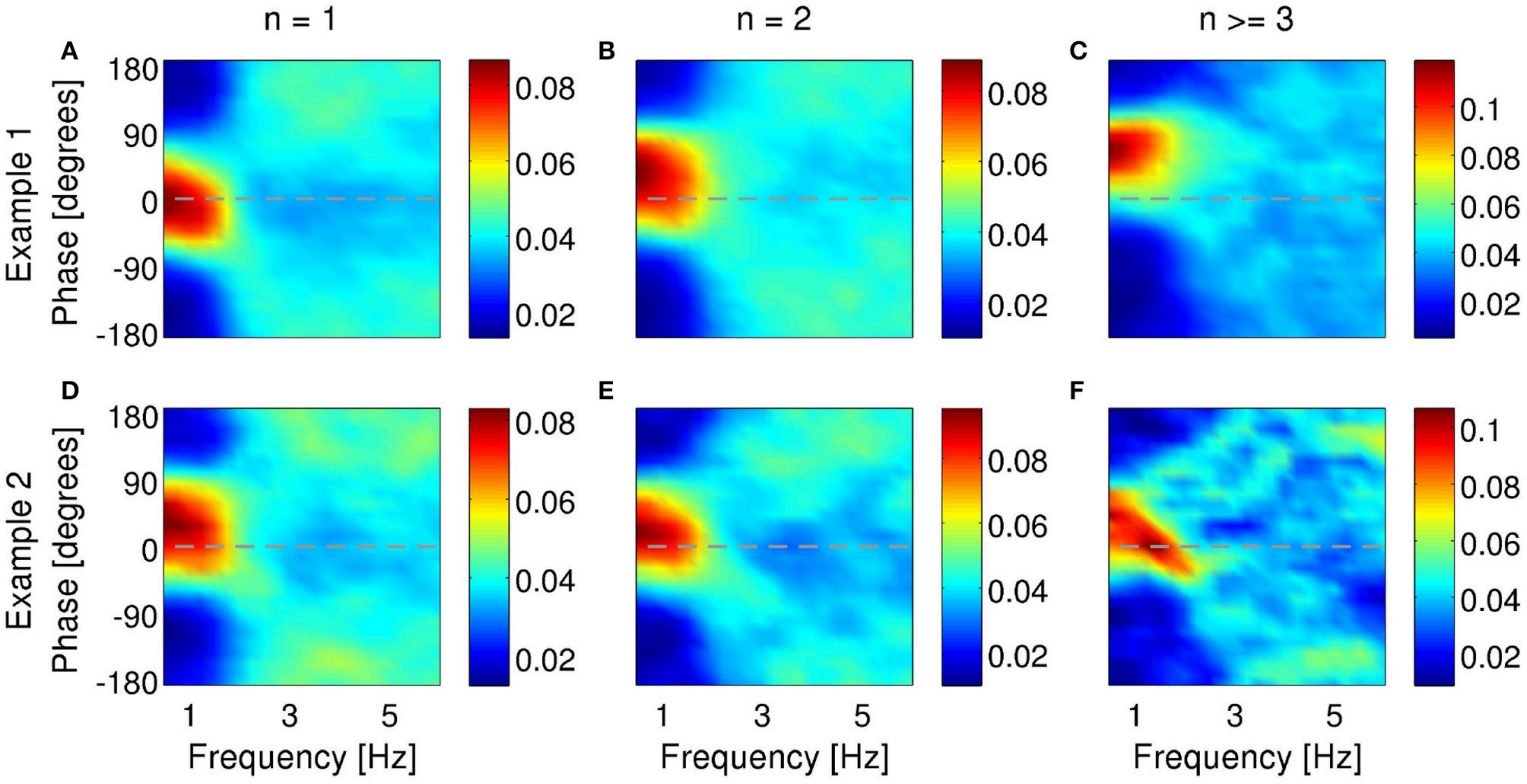
**Examples of phase locking of two bursting units (A–C)** and **(D–F)** identified in the subiculum of anesthetized rats when delta rhythms were dominant in the LFP. Phase-locking histograms of single spikes **(A,D)**, two-spike bursts **(B,E)** and larger bursts **(C,F)** fired by the example bursting units. Phase of 0^o^ indicates the peak of an oscillation. Colorbar: probability of firing *n*-spike bursts within a phase bin of narrowband-filtered LFP. Chance probability is equal to 0.04.

**Figure 9:**
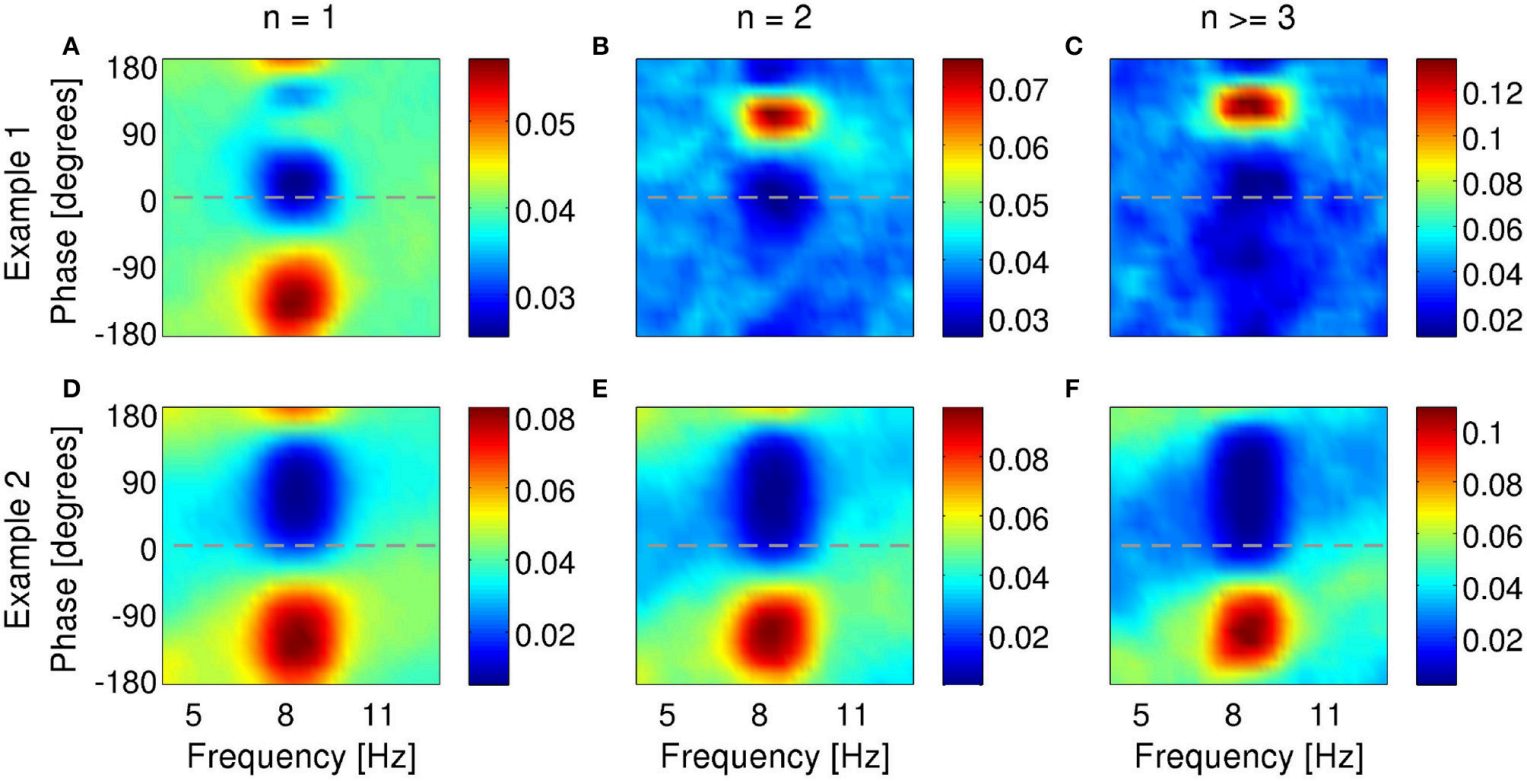
**Examples of phase locking of two bursting units (A–C)** and **(D–F)** identified in the MEC of awake behaving rats when theta rhythms were dominant in the LFP. Phase-locking histograms of single spikes **(A,D)**, two-spike bursts **(B,E)** and larger bursts **(C,F)** fired by the example bursting units. Phase of 0^o^ indicates the peak of an oscillation. Colorbar: probability of firing *n*-spike bursts within a phase bin of narrowband-filtered LFP. Chance probability is equal to 0.04.

For a neuron to transmit information about the phase of the LFP, bursting probability (with or without distinction of different *n*-values) must be modulated by the phase of the LFP. Under anesthesia, 61% of bursting units in subiculum locked their firing to a preferred phase of the delta-filtered LFP (examples in Figure 8). Sometimes, the preferred phase of locking shifted to more advanced or earlier phases as intra-burst spike count increased (36 and 4% of all bursting units, respectively). Figures 8A–C shows an example where the preferred phase of locking shifted from 0° to 90° with increasing burst size. This is the same cell as in Figures 3D–F, 7A. Not all cells displayed shifts, see for example Figures 8D–F. Two additional examples from the same dataset are shown in the supplementary data of Constantinou et al. (2015). Cells that are locked to a specific phase value for all burst sizes encode information about phase in the burst rate. Instead, cells whose preferred phase depends on *n* also encode information in the distinction between different *n*-values. In MEC, 59 % of bursting units locked their firing to a preferred phase of the theta rhythm; 12% of neurons exhibited a phase locking that shifted to more advanced phases, whereas 21% shifted to earlier phases. Two examples from the MEC are shown in Figure 9. Both cells locked to a preferred phase range of the theta-filtered LFP. For the first cell (Figures 9A–C), the preferred phase of locking shifted with increasing burst size. This shift was not observed in the second example (Figures 9D–F; same unit as in Figure 7B), implying that the phase was hardly encoded in burst size.

### 3.6. Bursting neurons encode features of dominant LFP rhythm

So far, we have examined the ability of bursting neurons to encode features of the dominant frequency band within the LFP: the delta band in the anesthetized animals and the theta band during exploratory behavior. However, neurons are immersed in a broadband LFP, so in principle, they could also encode features of more than a single frequency band. To verify whether such is the case, we narrowband-filtered the LFP over a range of frequencies and repeated the information analysis for each band.

**Figure 10:**
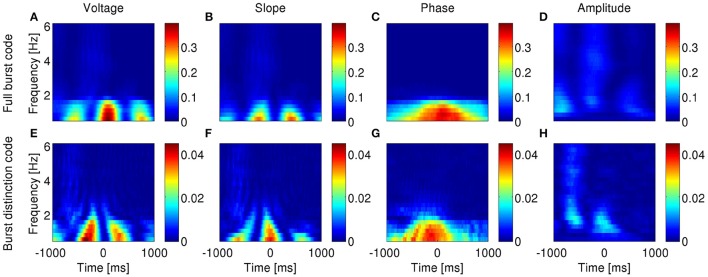
**Information encoded by bursting neuron output about LFP features as a function of LFP frequency and time around burst onset**. Example from a bursting unit in the rat subiculum during dominant delta rhythms under anesthesia. Information about the instantaneous voltage **(A,E)**, slope **(B,F)**, phase **(C,G)**, and amplitude **(D,H)** of narrowband-filtered LFP conveyed by the full burst code **(A–D)** and burst distinction code **(E–H)**. Colorbar: mutual information in bits/burst.

**Figure 11:**
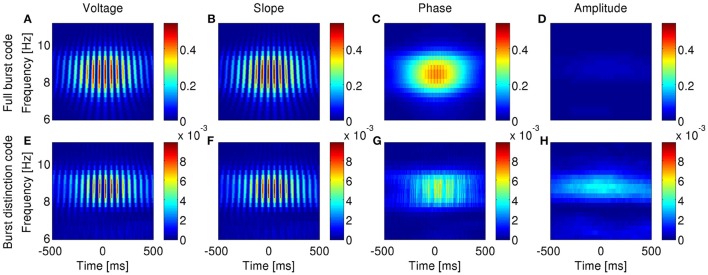
**Information encoded by bursting neuron output about LFP features as a function of LFP frequency and time around burst onset**. Example from a bursting unit in the rat MEC during awake behavior when theta rhythms were prevalent in the LFP. Information about the instantaneous voltage **(A,E)**, slope **(B,F)**, phase **(C,G)**, and amplitude **(D,H)** of narrowband-filtered LFP conveyed by the full burst code **(A–D)** and burst distinction code **(E–H)**. Colorbar: mutual information in bits/burst.

In agreement with model prediction (Supplementary Figures 5, 6), most subicular and all entorhinal neurons that encoded features of the band-filtered LFP showed maximal information encoding in the frequency band with highest power but not other frequencies (examples in Figures 10, 11). Five of the encoding subicular cells also showed information encoding of the instantaneous amplitude of LFP at frequencies higher than ~6 Hz (example in Supplementary Figure 7). The information about voltage and slope exhibited the same oscillatory patterns observed in Figures 3, 4 with the frequency of the oscillations being twice the frequency at which the signal was filtered. The oscillations in information therefore became narrower as the frequency increased. The information encoded by the burst rate code was very similar to that of the full burst code.

## 4. Discussion

Bursts encode behaviorally-relevant information in several systems (Guido and Weyand, 1995; Sherman, 2001; Swadlow and Gusev, 2001; Chacron et al., 2004; Lesica and Stanley, 2004; Oswald et al., 2004; Marsat and Pollack, 2006; Sabourin and Pollack, 2009). In particular, temporally-structured neural codes have been found to encode information both in the timing and the spike count of bursts (DeBusk et al., 1997; Martinez-Conde et al., 2002; Arganda et al., 2007; Eyherabide et al., 2008, 2009; Marsat and Pollack, 2010). Neurons in the hippocampal formation are equipped with the endogenous mechanisms required for bursting (Hablitz and Johnston, 1981; Taube, 1993) and are tightly regulated by inhibitory networks that modulate bursting (Royer et al., 2012). Moreover, neurons are immersed in strongly oscillating fields that may favor temporally structured outputs such as bursting (Mizuseki et al., 2009). Therefore, bursts are likely to subserve a number of computational functions. For example, bursts generated at different frequencies induce long-term potentiation involving different ionic mechanisms and lasting different time intervals (Grover et al., 2009). Bursts are also involved in replay sequences during slow wave sleep (Lee and Wilson, 2002) and REM sleep (Louie and Wilson, 2001). It is therefore important to determine the contextual conditions in which bursts are generated, in particular, the statistical relation between the surrounding LFP and burst initiation.

We found that the probability of generating a burst of *n* spikes decreased with *n*, showing a steeper decay for the awake data. A large fraction of bursting cells encoded significant amounts of information about at least one of the tested features (instantaneous voltage, slope, phase and amplitude), even though cells were only selected according to their ISI histogram. No criterion regarding neuronal type was used to exclude cells. In the MEC, the number of informative neurons was smaller than in the subiculum, but the informative neurons encoded more information. Spikes belonging to the same burst often decrease progressively in amplitude (Kandel and Spencer, 1961; Ranck, 1973), and could thus be assigned to different cells by typical spike sorting techniques (Harris et al., 2000). Therefore, our experimental results constitute a lower bound to the burst-mediated code, since there are potentially more bursts in the data than the ones we detected.

In the codes we studied, all bursts of *n* spikes were described by the same symbol (indicating the spike count in the full burst and burst distinction codes or the occurrence of a burst in the burst rate code) assuming that small differences in the ISI inside the burst are uninformative. As a result, the space of all possible spike patterns is reduced to a much smaller space, in which only burst-like patterns matter. The reduction could, in principle, discard information, because the neural code is not guaranteed to occur by means of a discrete alphabet (Eyherabide and Samengo, 2010a,b). The advantage, however, is that information measures do not require the study of long response windows, and by studying a small number of BTAs, the neural code is revealed.

The timing of each burst was defined as the time of the first spike in the burst. In principle, other choices could have been considered, such as the last spike or the mid-point. Since the investigated burst codes only make sense if all bursts of the same duration are taken as the same symbol (fluctuations in the duration are neglected), shifting the time assigned to each burst is an invertible transformation, so the data processing inequality reduces to an equality. Therefore, the mutual information values remain unchanged. The only difference is that the value of information, which we now assign to time *t*, would be assigned to time *t* − *t*_shift_, and the same would happen to BTAs. The shift would therefore displace the graphs, but the conclusions of the paper would still be valid.

We found that most of the information about the LFP was encoded in the timing of burst initiation, implying that the code mainly represented temporal information. Burst onset punctuated LFP features falling within a specific range. Some cells also encoded 10–15% of additional information in the differentiation between bursts of different spike counts. The additional information represented fine-grained distinctions between the encoded feature values.

In the MEC, most cells encoded voltage, phase and slope simultaneously, and an independent subset of cells encoded amplitude. In the subiculum, most cells encoded a large amount of information about voltage and phase, and approximately half that amount about slope and amplitude. In order to understand these correlations, it is important to notice that the four tested features are not independent from one another. The LFP contains temporal correlations, and therefore induces a certain amount of statistical dependence between voltage, slope, phase and amplitude. Both in the theta and the delta-dominated LFPs, phase was correlated with voltage. The mutual information between the two features was approximately 0.8–1 bit (out of a maximum of 2 bits, given the employed binning). Phase and slope were less correlated and the mutual information between them was approximately 0.5 bits out of 2. Amplitude was mildly correlated with voltage in the delta-dominated LFP (mutual information was 0.3 bits out of 2), and even less in the theta-dominated LFP (mutual information was 0.1 bits out of 2). Importantly, slope was not correlated with voltage (mutual information was less than 0.1 bit out of 2), and by construction, phase and amplitude were independent. Therefore, the high correlation between the information encoded by bursting neurons about voltage, phase and slope found in the MEC could be potentially explained by an encoding mechanism mainly focused on representing phase, the other two features being no more than residual epiphenomena. There is no single feature whose encoding can explain the results found in the subiculum, so we must either conclude that at least two features are encoded (for example, voltage and slope, or phase and amplitude), or that a yet unexplored feature plays the protagonist role.

Although there is no complete understanding of the mechanisms through which the LFP arises, many authors agree that the main contribution is provided by the extracellular currents produced by synaptic input to a given brain region (Logothetis, 2003; Buzsáki et al., 2012; Einevoll et al., 2013). Hence, LFP fluctuations mainly reflect fluctuations in the input, the output activity of the local neurons playing only a minor role. It may therefore be puzzling to find that bursts also encode future LFP values, which seems to violate causality. It should be noticed, however, that such future encoding is also found in the simulations, where by construction, neural activity is the consequence (and not the cause) of the driving signal. As discussed in Samengo et al. (2013), encoding of future input features only takes place in signals that contain temporal correlations themselves. One can only expect a burst to encode future stimulus values if the burst is driven by input currents whose present value contains information about how they will evolve in the near future. Therefore, predictive encoding is only expected to occur up to time windows that are within the range of the temporal correlations of the signal itself. Indeed, we found that when the LFP is dominated by theta, bursts can predict features that extend up to 250 ms into the future, that is 1–2 theta cycles. Instead, for delta-dominated LFPs, the encoding goes as far as 500 ms, again, 1–2 cycles of the much slower delta.

The computational model used to simulate bursting neurons was able to reproduce the main results obtained with the experimental data. The model contained the minimal ionic conductances required for inducing bursting and thus, by construction, does not represent every biophysical detail that generates bursts in all real neurons. Even so, the simulations are useful to show that the differences observed in the neural code of behaving and anesthetized animals can be obtained by simply changing the frequency content and the amplitude of the driving signal, rather than the specific biophysical mechanisms of a particular bursting neuron.

In summary, we have combined computational modeling with analysis of *in-vivo* data from awake and anesthetized rats with the aim to determine the code by which burst firing in the hippocampal formation can convey information about features of ongoing LFP oscillations. Our results confirm that the burst code represents the temporal features of the predominant frequency band of the extracellular oscillations, and that most of the information is encoded in the timing of burst onset. A more complex code, in which the different burst sizes are distinguished, added a further 10–15% of information. These findings suggest that bursts may have an important role in relaying information encoded in the LFP to downstream neurons.

We interpret the term “information” in the technical sense defined by Shannon: the reduction in uncertainty about the value of a LFP feature by observing the response of the bursting neuron. This interpretation of the word information follows the line of the classical studies in the topic, as for example by Rieke et al. (1997); Borst and Theunissen (1999); Quian Quiroga and Panzeri (2009). We do not, however, address the issue of whether or how this encoding is further exploited by the brain. However, this does not preclude us to hypothesize about its possible function. Theta and delta rhythms are known to be involved in processing information related to declarative memory. In addition, previous studies demonstrated that the information carried by spikes is boosted by knowledge of LFP features (Montemurro et al., 2008; Kayser et al., 2009). After Samengo and Montemurro (2010), the hypothesis that bursting could be involved in making such information available to downstream neurons became more credible. Our current paper, then, is the first to actually show that the hippocampal formation is indeed endowed with the mechanisms to do so. We hope to motivate other scientists to search for evidence relating to the decoding of this information by downstream neurons and also whether these mechanisms are present in other regions.

## Author contributions

MC, SGC, and IS analyzed data. MC, SGC, IS, and MM wrote code. MC and DE programmed the simulations. EK and JG collected the data. IS and MM designed the study. MC, SGC, IS, and MM wrote the paper. MC wrote the supplementary material. All authors proofread the manuscript.

## Funding

MC was funded by a Doctoral Training Partnership PhD Studentship awarded to the University of Manchester by the UK Biotechnology and Biological Sciences Research Council (BBSRC DTP grant code: BB/J014478/1) and a President's Doctoral Scholar Award by the University of Manchester. SGC and IS were supported by Consejo Nacional de Investigaciones Científicas y Técnicas (grant code: PIP 11220090100738) and Universidad Nacional de Cuyo. SGC, EK, IS, and MM were supported by Proyecto Raíces Siembra of Agencia Nacional de Promoción Científica y Tecnológica.

### Conflict of interest statement

The authors declare that the research was conducted in the absence of any commercial or financial relationships that could be construed as a potential conflict of interest.
