# Supplementary material for: Bursting Neurons in the Hippocampal Formation Encode Features of LFP Rhythms
Source: Front Comput Neurosci. 2016 Dec 26;10:133. doi: 10.3389/fncom.2016.00133 (PMC5183636; doi:10.3389/fncom.2016.00133)
Supplement: Supplementary file 1 [file DataSheet1.pdf]

Supplementary Material: Bursting Neurons in the Hippocampal Formation Encode Features of LFP Rhythms

Maria Constantinou *, Soledad Gonzalo Cogno , Daniel H. Elijah , Emilio Kropff , John Gigg , Inés Samengo and Marcelo A. Montemurro
*Correspondence:
Maria Constantinou: Faculty of Biology, Medicine and Health, The University of Manchester, Oxford Road, Manchester, M13 9PT, UK
maria.constantinou@outlook.com

1 SUPPLEMENTARY METHODS

1.1 Bursting neuron model

The equations and parameters of the bursting neuron model have been published before in Constantinou et al. (2015) and are included here for completion. The model consisted of two compartments: dendrites and soma. The input signal $I(t)$ was injected in the dendritic compartment and bursting activity was recorded from the somatic compartment as described in Supplementary Equations 1 and 2, respectively.

$$C_m \frac{dV_d}{dt} = -I_L - I_{KS} - I_{NaP} - g_c \frac{V_d - V_s}{1-p} + I(t) \tag{1}$$

$$C_m \frac{dV_s}{dt} = -I_L - I_K - I_{Na} - g_c \frac{V_s - V_d}{p} \tag{2}$$

The equations of the ionic currents and the model parameters are listed in Supplementary Tables 1 and 2, respectively. The 4th order Runge-Kutta method with 0.01 ms time step was used for numerical integration of the model.

1.2 Discretization of LFP feature signals

LFP features (voltage, slope, phase and amplitude) vary continuously. However, estimating the probability of continuous LFP features from a finite sample would result in an enormous bias. Hence, the continuous signal was discretized into a finite number of bins M chosen by optimizing the trade-off between being large enough to preserve most of the information and, at the same time, small enough to reduce the bias, as in Elijah et al. (2015). In short, we varied M and, for each value, obtained the feature set X, from which we computed the mutual information $I(X; N) = I(X) - I(X|N)$, the bias estimate $I_s(X; N) = \langle I(X) - I(X|N_s) \rangle$ (where $\langle ... \rangle$ indicates average over 100 repetitions), and the bias-corrected information $I_c(X; N) = I(X; N) - I_s(X; N)$. We computed these measures for the burst distinction code with no time lag, so that $N = \{1, 2, 3\}$ and X comprised the features occurring at the time of burst onset.

The results were similar for all bursting neurons (example in Supplementary Figure 4). When $M < 2^2$, the information measures were underestimated; whereas when $M > 2^3$, there was considerable bias in the information estimate $I(X; N)$ as depicted by the diverging lines of $I(X; N)$ and the bias-corrected

information estimate $I_c(X;N)$. The bias correction method we used was sufficient to correct for this as indicated by the plateau of $I_c(X;N)$ (Supplementary Figure 4). Since there was no considerable gain in information by using $M > 2^2$, LFP features were discretized with $M = 4$.

1.3 Relation between the three burst codes

This section explains how we used the chain rule for mutual information to derive the equation $I(X;\{0,1,2,3\}) = I(X;\{0,\text{burst}\}) + r\delta t I(X;\{1,2,3\})$ which relates the three codes we investigated.

In the full burst code: $N = \{0,1,2,3\}$, in the burst rate code: $N = \{0,\text{burst}\}$, and in the burst distinction code: $N = \{1,2,3\}$. We define the variables

$$\begin{aligned} W &= \{0,1,2,3\}, \\ Y &= \{0,\text{burst}\}, \\ Z &= \{1,2,3\}. \end{aligned} \tag{3}$$

Since the three variables arise from the same neural response, Y is a deterministic function of W,

$$Y(W) = \begin{cases} 0 & \text{if } W = 0, \\ \text{burst} & \text{if } W \neq 0. \end{cases} \tag{4}$$

When $Y = \text{burst}$, the variable Z is also a deterministic function of W,

$$Z(W) = \begin{cases} W & \text{if } W > 0, \\ \text{not defined} & \text{if } W = 0. \end{cases} \tag{5}$$

The chain rule states that for any three variables X, A, B,

$$I(X;A,B) = I(X;A) + I(X;B|A). \tag{6}$$

If we take $A = W$ and $B = Y$, the chain rule becomes

$$I(X;W,Y) = I(X;W) + I(X;Y|W). \tag{7}$$

The deterministic rule of Supplementary Equation 4 implies that $H(Y|W) = 0$, so Y cannot transmit information about any variable, when conditioned on W. That is, $I(X;Y|W) = 0$. Hence, Supplementary Equation 7 reduces to

$$I(X;W,Y) = I(X;W). \tag{8}$$

If we now take $A = Y$ and $B = W$, the chain rule becomes

$$I(X;Y,W) = I(X;Y) + I(X;W|Y). \tag{9}$$

In addition,

$$\begin{aligned} I(X;W|Y) &= P(y=0) \sum_{x \in X} \sum_{w \in W} P(x,w|y=0) \log \frac{P(x,w|y=0)}{P(x|y=0)P(w|y=0)} + \\ &+ P(y=\text{burst}) \sum_{x \in X} \sum_{w \in W} P(x,w|y=\text{burst}) \log \frac{P(x,w|y=\text{burst})}{P(x|y=\text{burst})P(w|y=\text{burst})} \end{aligned} \tag{10}$$

Supplementary Equation 4 implies that when $y = 0$, there is no alternative but to have $w = 0$. Therefore, in the first term of Supplementary Equation 10, only the term with $w = 0$ appears, for all others, $P(x, w|y = 0)$ vanishes. Moreover, $P(x, w = 0|y = 0) = P(x|y = 0)$, so the first term vanishes. Similarly, Supplementary Equation 5 implies that when $y = \mathrm{burst}$, w is equal to z, and $P(x, w|y = \mathrm{burst}) = P(x, z)$. Therefore, Supplementary Equation 9 becomes

$$I(X; Y, W) = I(X; Y) + P(Y = \mathrm{burst})I(X; Z). \tag{11}$$

Given that $P(Y = \mathrm{burst}) = r\delta t$, and taking Supplementary Equation 8 into account, we get

$$I(X; W) = I(X; Y) + r\delta t\, I(X; Z). \tag{12}$$

2 SUPPLEMENTARY RESULTS

Theta rhythms can be separated in two types based on their sensitivity to atropine (Kramis et al., 1975). Urethane preserves the atropine-sensitive theta (3-7 Hz) but eliminates the atropine-resistant theta (7-12 Hz) (Kramis et al., 1975; Clement et al., 2008). We observed that, under urethane anesthesia, the LFP of three rats exhibited shifts in which either delta ($\sim$1 Hz) or theta rhythms ($\sim$4 Hz) were dominant (example in Supplementary Figure 2). We identified 13 bursting units during the epochs containing dominant theta rhythms under anesthesia. These units were also bursting during epochs of dominant delta rhythms. We analyzed these data separately for the theta-dominant epochs and present the results here.

We investigated whether these cells encoded features of theta rhythms in their bursting output. Eleven cells showed evidence of encoding the instantaneous voltage, slope and phase of theta rhythms by the full burst code and the burst rate code (examples in Supplementary Figures 8A-B,D-E and 9A-D); and five of these cells also encoded the instantaneous amplitude. For the latter cells, the information encoded for voltage, slope and phase was twice to ten times higher than of amplitude. Four of the encoding cells also showed evidence of feature encoding by the burst distinction code (example in Supplementary Figures 8C and 9E-H). One cell encoded most information about the instantaneous amplitude of theta rhythms, and less about the voltage and slope, by the full burst code and burst rate code. These results suggest that bursting neurons can encode information conveyed by atropine-sensitive theta rhythms during anesthesia.

REFERENCES

Clement, E. A., Richard, A., Thwaites, M., Ailon, J., Peters, S., and Dickson, C. T. (2008). Cyclic and sleep-like spontaneous alternations of brain state under urethane anaesthesia. *PLoS ONE* 3, e2004. doi:10.1371/journal.pone.0002004

Constantinou, M., Elijah, D. H., Squirrell, D., Gigg, J., and Montemurro, M. A. (2015). Phase-locking of bursting neuronal firing to dominant LFP frequency components. *BioSystems* 136, 73–79. doi:10.1016/j.biosystems.2015.08.004

Elijah, D. H., Samengo, I., and Montemurro, M. A. (2015). Thalamic neuron models encode stimulus information by burst-size modulation. *Front. Comput. Neurosci.* 9, 113. doi:10.3389/fncom.2015.00113

Kramis, R., Vanderwolf, C. H., and Bland, B. H. (1975). Two types of hippocampal rhythmical slow activity in both the rabbit and the rat: relations to behavior and effects of atropine, diethyl ether, urethane, and pentobarbital. *Exp. Neurol.* 49, 58–85. doi:10.1016/0014-4886(75)90195-8

3 SUPPLEMENTARY TABLES AND FIGURES

Supplementary Table 1. Equations describing the ionic currents of the two-compartment model. The last row shows the kinetics equation of the gating variables for the Na, K and slow K currents.

Na current	$I_{Na} = g_{Na} m_\infty^3 h(V_s - E_{Na})$<br>$m_\infty = \alpha_m/(\alpha_m + \beta_m)$<br>$\alpha_m = -0.1(V_s + 31)/(\exp(-0.1(V_s + 31)) - 1)$<br>$\beta_m = 4\exp(-(V_s + 56)/18)$<br>$\alpha_h = 0.07\exp(-(V_s + 47)/20)$<br>$\beta_h = 1/\exp(-0.1(V_s + 17)) + 1)$
K current	$I_K = g_K n^4 (V_s - E_K)$<br>$\alpha_n = -0.01(V_s + 34)/(\exp(-0.1(V_s + 34)) - 1)$<br>$\beta_n = 0.125\exp(-(V_s + 44)/80)$
Persistent Na current	$I_{NaP} = g_{NaP} r_\infty^3 (V_d - E_{Na})$<br>$r_\infty = 1/(\exp(-(V_d + 57.7)/7.7) + 1)$
Slow K current	$I_{KS} = g_{KS} q(V_d - E_K)$<br>$q_\infty = 1/(\exp(-(V_d + 35)/6.5) + 1)$<br>$\tau_q = \tau_{q_0}/(\exp(-(V_d + 55)/30) + \exp((V_d + 55)/30))$, $\tau_{q_0} = 200$
Leak currents	$I_L = g_L(V - E_L)$, where $V = V_d$ or V_s
Kinetics of gating variables	$dx/dt = \phi_x(\alpha_x(1 - x) - x\beta_x) = \phi_x(x_\infty - x)/\tau_x$, where $x = h$, n or q

Supplementary Table 2. Parameters of the two-compartment model.

Reversal potentials (mV)	E_{Na}<br>E_K<br>E_L	55<br>-90<br>-65
Maximum conductances (mS/cm^2)	g_{Na}<br>g_K<br>g_L<br>g_{NaP}<br>g_{KS}	45<br>15<br>0.18<br>0.08<br>0.7
Coupling conductance (mS/cm^2)	g_c	1
Membrane capacitance (μF/cm^2)	C_m	0.6
Relative area between compartments	p	0.15
Temperature scaling factors	ϕ_h<br>ϕ_n<br>ϕ_q	3.33<br>3.33<br>1

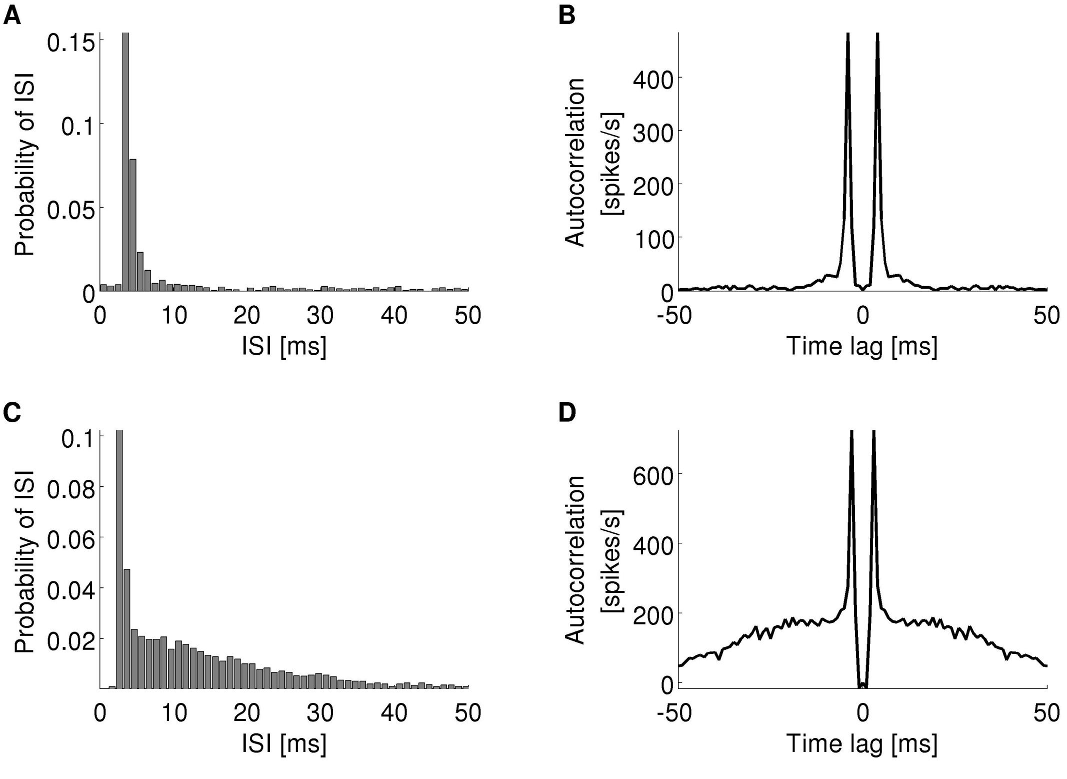

Supplementary Figure 1. Example of ISI histograms **(A,C)** and autocorrelograms **(B,D)** of a busting unit in the subiculum **(A,B)** and the MEC **(C,D)**. A unit was classified as bursting if there was a sharp peak within 2-8 ms for subiculum **(A,B)** or 2-5 ms for MEC **(C,D)** in the autocorrelogram and ISI histogram but not another peak within 50 ms.

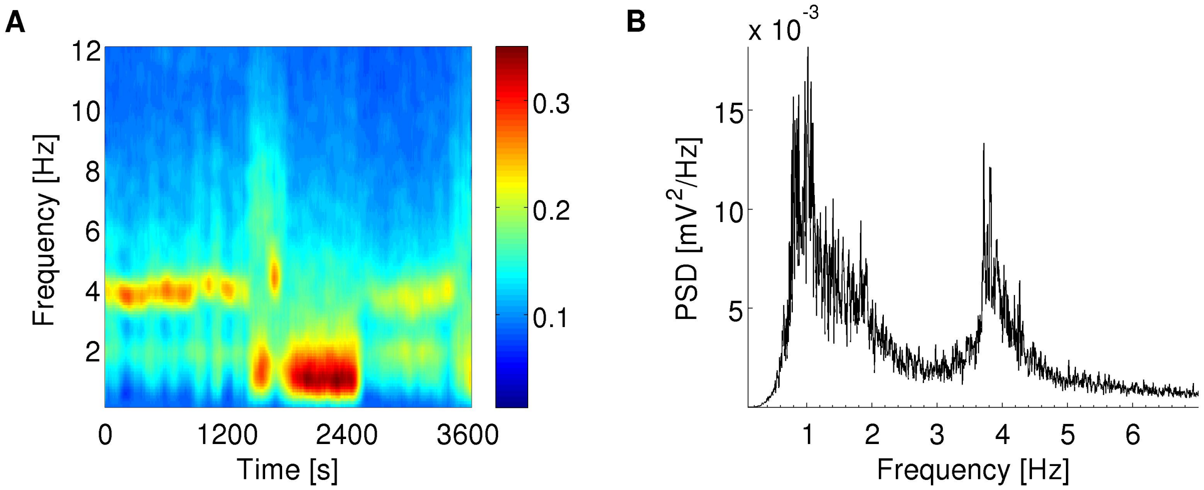

Supplementary Figure 2. Example of spectrogram **(A)** and power spectrum **(B)** of the LFP recorded by an electrode in the subiculum of a urethane-anesthetized rat. The LFP shifts between states in which there is a spectral peak at either ∼1 Hz or ∼4 Hz. [**A**: The color scale is in $(mV^2/Hz)^{0.25}$. Warmer colors indicate higher power spectral density.]

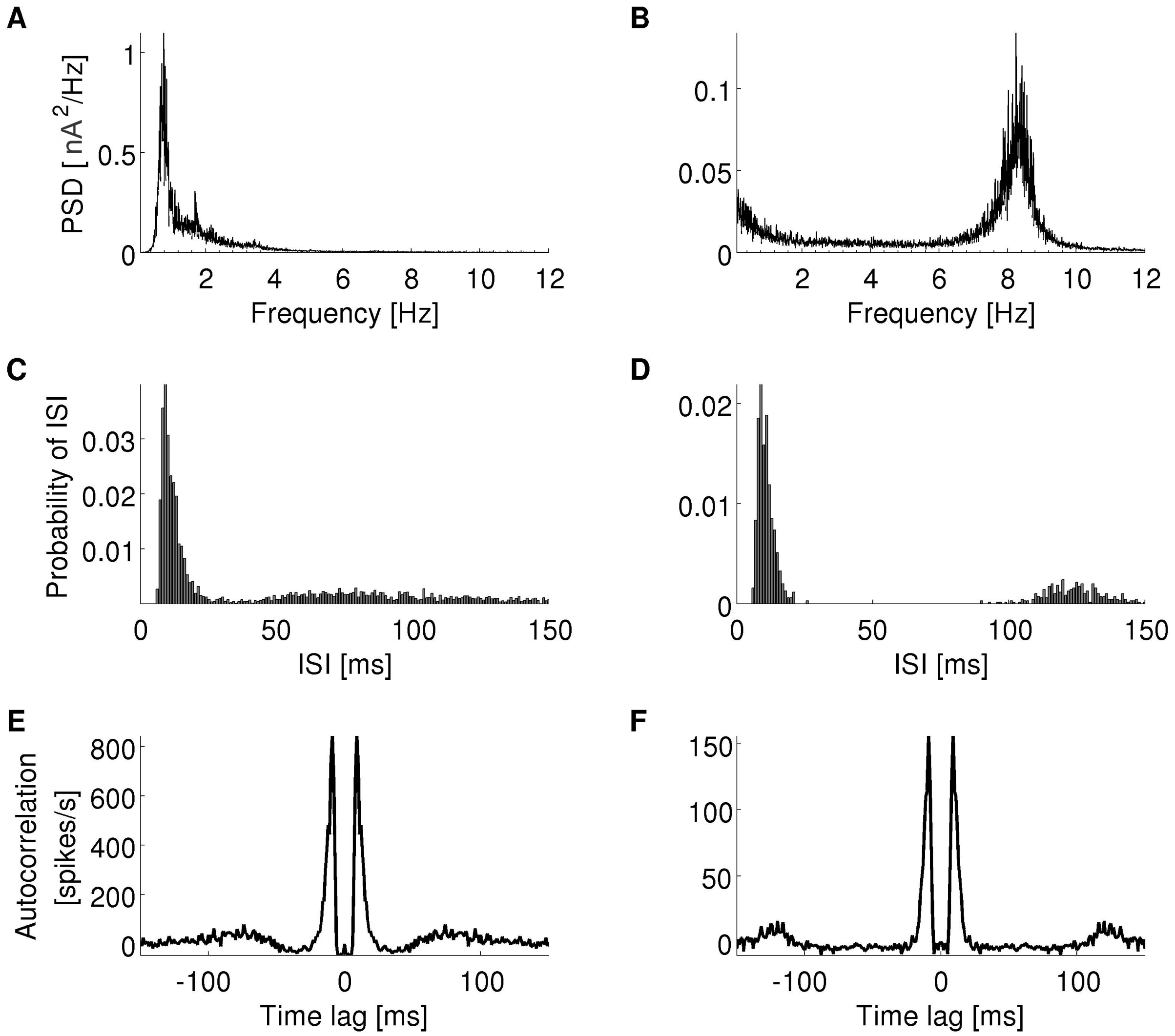

Supplementary Figure 3. Power spectra of input signals to the model containing peaks at delta rhythms **(A)** or theta rhythms **(B)**. ISI histograms **(C,D)** and autocorrelograms **(E,F)** of the spiking output of the model when the input had the corresponding spectra in **A,B**.

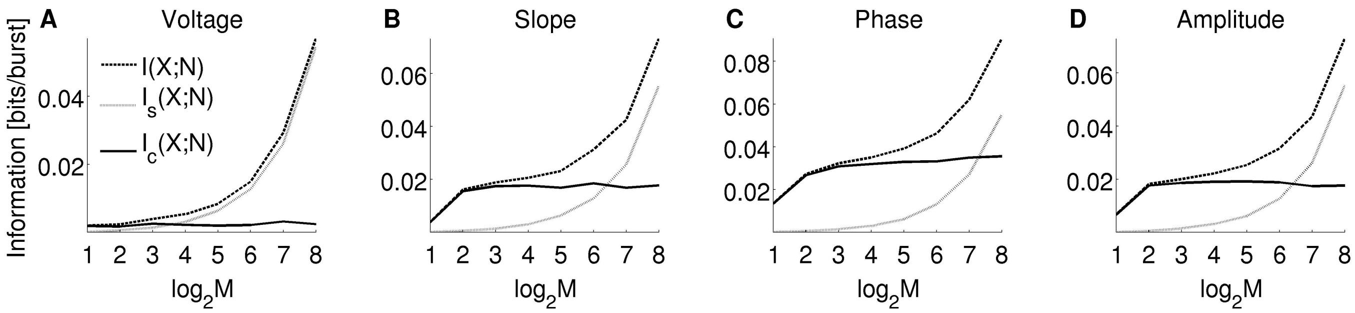

Supplementary Figure 4. Information and bias estimates as a function of the number of bins M used to discretize the instantaneous voltage **(A)**, slope **(B)**, phase **(C)** and amplitude **(D)** of the LFP. Information measures were estimated for the burst distinction code at the time of burst onset. This example is for the same unit as in Figure 3D-F.

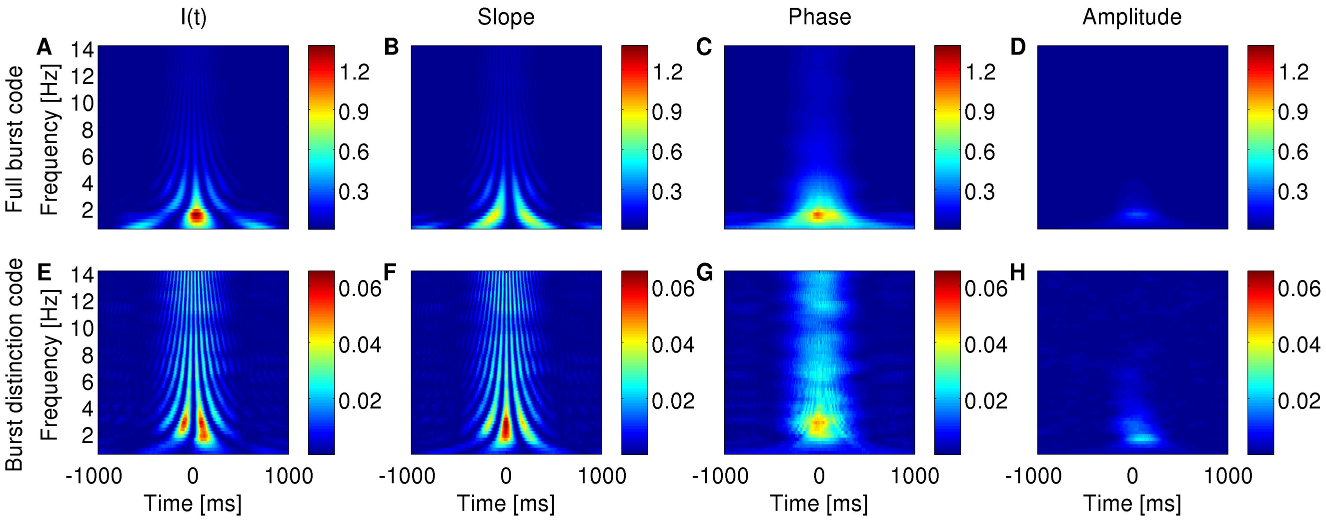

Supplementary Figure 5. Information encoded by the bursting neuron model about features of the input signal $I(t)$ as a function of frequency and time around burst onset. For these simulations, $I(t)$ contained dominant delta rhythms to mimic the LFP rhythms present during anesthesia. Information about the instantaneous $I(t)$ **(A,E)**, slope **(B,F)**, phase **(C,G)** and amplitude **(D,H)** of narrowband-filtered $I(t)$ conveyed by the full burst code **(A-D)** and burst distinction code **(E-H)**. Colorbar: mutual information in bits/burst.

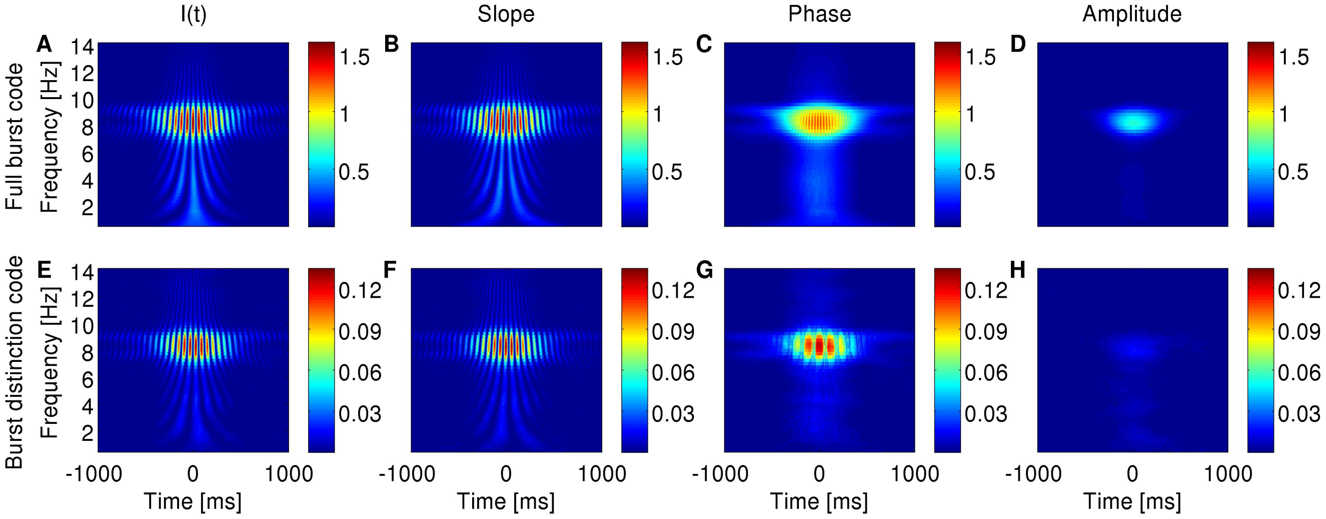

Supplementary Figure 6. Information encoded by the bursting neuron model about features of the input signal $I(t)$ as a function of frequency and time around burst onset. For these simulations, $I(t)$ contained dominant theta rhythms to mimic the LFP rhythms present during exploratory behavior. Information about the instantaneous $I(t)$ **(A,E)**, slope **(B,F)**, phase **(C,G)** and amplitude **(D,H)** of narrowband-filtered $I(t)$ conveyed by the full burst code **(A-D)** and burst distinction code **(E-H)**. Colorbar: mutual information in bits/burst.

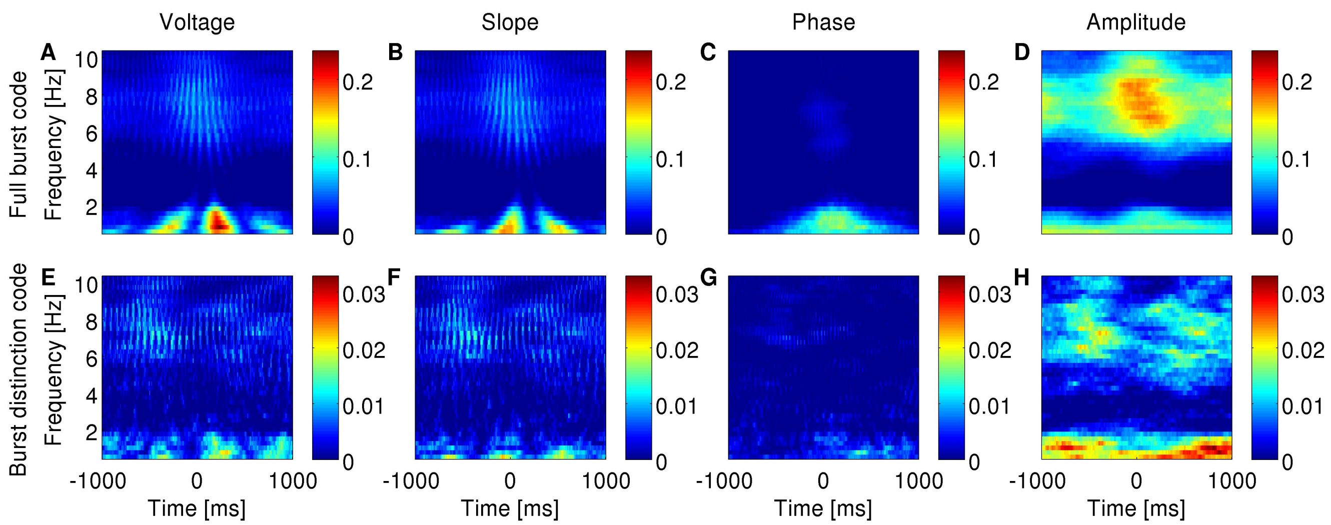

Supplementary Figure 7. Information encoded by bursting neuron output about LFP features as a function of LFP frequency and time around burst onset. Example from a bursting unit in the rat subiculum during dominant delta rhythms under urethane-anesthesia. Information about the instantaneous voltage **(A,E)**, slope **(B,F)**, phase **(C,G)** and amplitude **(D,H)** of narrowband-filtered LFP conveyed by the full burst code **(A-D)** and burst distinction code **(E-H)**. Colorbar: mutual information in bits/burst.

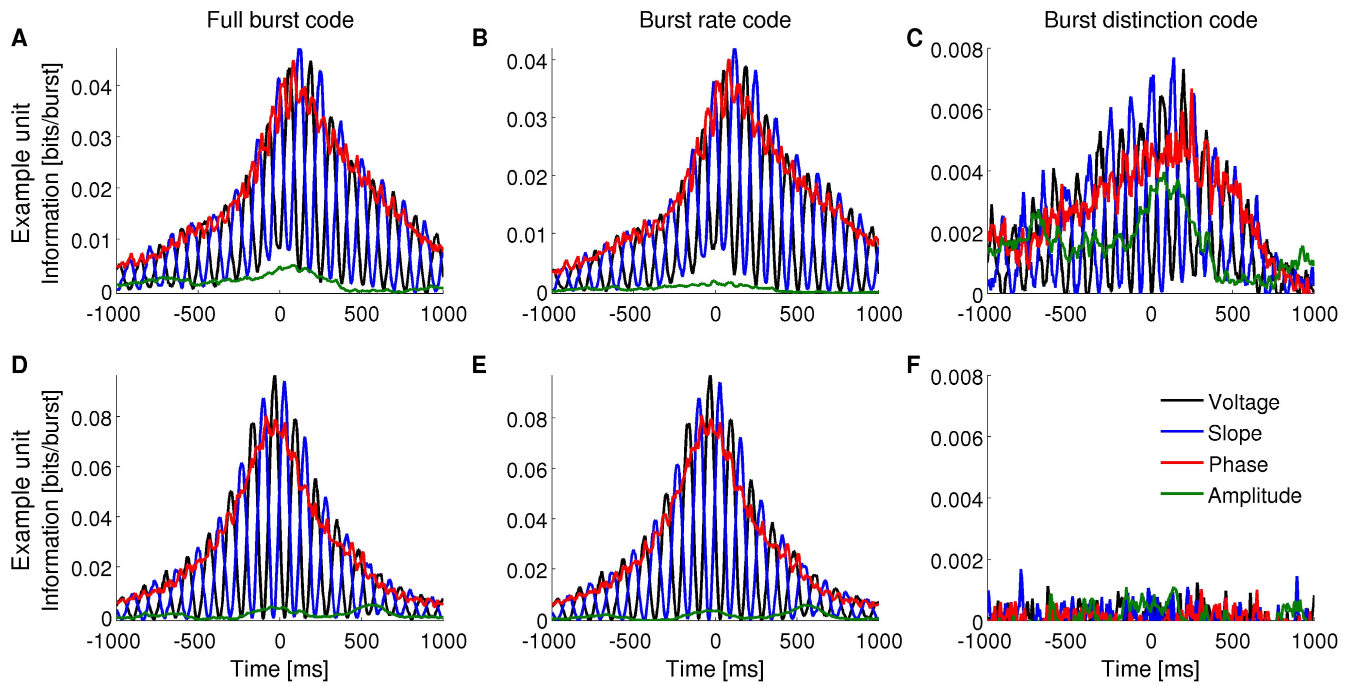

Supplementary Figure 8. Information encoded by bursting output of two different subicular cells **(A-C** and **D-F)** about the voltage, slope, phase and amplitude of theta rhythms in the LFP during urethane-anesthesia. Both cells encode information about the voltage, slope and phase of theta-filtered LFP by the full burst code **(A,D)** and burst rate code **(B,E)**. One of the cells encodes information about LFP features in the distinction between different burst sizes **(C)** whereas the second does not **(F)**. LFPs were filtered within 2.5-5 Hz. x-axis: time relative to burst onset defined at 0 ms.

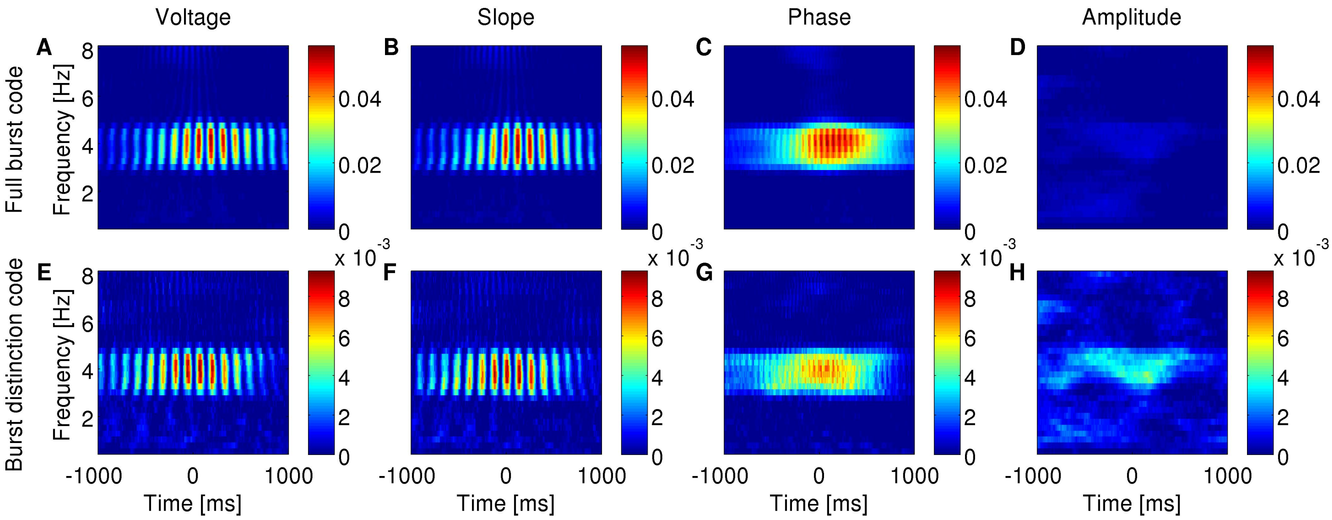

Supplementary Figure 9. Information encoded by bursting neuron output about LFP features as a function of LFP frequency and time around burst onset. Example from a bursting unit in the rat subiculum during dominant theta rhythms under urethane-anesthesia. Information about the instantaneous voltage **(A,E)**, slope **(B,F)**, phase **(C,G)** and amplitude **(D,H)** of narrowband-filtered LFP conveyed by the full burst code **(A-D)** and burst distinction code **(E-H)**. Colorbar: mutual information in bits/burst.
